# Stress-induced and epigenetic-mediated maize transcriptome regulation study by means of transcriptome reannotation and differential expression analysis

**DOI:** 10.1038/srep30446

**Published:** 2016-07-27

**Authors:** Cristian Forestan, Riccardo Aiese Cigliano, Silvia Farinati, Alice Lunardon, Walter Sanseverino, Serena Varotto

**Affiliations:** 1 Department of Agronomy, Animals, Food, Natural Resources and Environment, University of Padova, Viale dell’Università 16, 35020 Legnaro (PD)-Italy; 2 Sequentia Biotech SL, Calle Comte D’Urgell, 240, Barcelona, Spain; 3 Department of Biology and Huck Institutes of the Life Sciences, Penn State University, University Park, PA 16802 USA

## Abstract

Plant’s response and adaptation to abiotic stresses involve sophisticated genetic and epigenetic regulatory systems. To obtain a global view of molecular response to osmotic stresses, including the non-coding portion of genome, we conducted a total leaf transcriptome analysis on maize plants subjected to prolonged drought and salt stresses. Stress application to both B73 wild type and the epiregulator mutant *rpd1-1*/*rmr6* allowed dissection of the epigenetic component of stress response. Coupling total RNA-Seq and transcriptome re-assembly we annotated thousands of new maize transcripts, together with 13,387 lncRNAs that may play critical roles in regulating gene expression. Differential expression analysis revealed hundreds of genes modulated by long-term stress application, including also many lncRNAs and transposons specifically induced by stresses. The amplitude and dynamic of the stress-modulated gene sets are very different between B73 and *rpd1-1*/*rmr6* mutant plants, as result of stress-like effect on genome regulation caused by the mutation itself, which activates many stress-related genes even in control condition. The analyzed extensive set of total RNA-Seq data, together with the improvement of the transcriptome and the identification of the non-coding portion of the transcriptome give a revealing insight into the genetic and epigenetic mechanism responsible for maize molecular response to abiotic stresses.

Plants are exposed to a variety of environmental stresses during their life cycle, with drought and salinity stresses representing the leading constraints to growth and productivity, being responsible for high yield loss and lower harvest quality in agriculture^1^,^2^. In particular, water scarcity accounts for about 70 per cent of potential yield losses worldwide, and in arid and semi-arid regions is coupled with salt-affected soils. It is thus fundamental to improve agricultural water use efficiency through the development of crop varieties with increased tolerance to drought and salinity^3^,^4^,^5^,^6^. However, the genetic control of tolerance to abiotic stresses is not only very complex, but is also strongly influenced by other environmental factors and by the developmental stage of the plant^7^. The plant’s response to these stresses is accompanied by the activation of many genes involved in stress perception and in transduction of the stress signal, which results in further modulation of gene expression^8^,^9^.

In the last years, the understanding of the plant’s molecular response to osmotic stress has greatly increased by means of deep sequencing application to transcriptomic profiling of a growing number of species^10^,^11^,^12^. RNA-Seq has great sensitivity and a high base resolution capacity to discriminate between splicing variants, alleles and sense/antisense isoforms, allowing the identification of the genome-wide mechanisms involved in plant stress response, adaptation and resilience^13^. Moreover, total RNA-Seq can capture the most complete picture of the transcriptome, including coding and multiple forms of non-coding RNA^14^. Some of them, called long non-coding RNAs (lncRNA) are recognised as an important component of development regulatory mechanisms involved in chromatin modification, epigenetic regulation, genomic imprinting, transcriptional control, as well as pre- and posttranslational mRNA processing. lncRNAs can be transcribed from intergenic regions or in antisense of canonical genes^15^.

Sequencing of plant total RNAs can also lead to the sequencing of transposable elements (TE) transcripts, and more specifically of those transcripts of TEs that can move around the genome via RNA intermediates, called retrotransposons^16^. In plants, TEs are normally transcriptionally silenced throughout DNA methylation at cytosines in every sequence context (mCG, mCHG, mCHH, where H represents an A, T or C), with methylation at lysine 9 of histone H3 (H3K9me2) and by 24nt sRNAs that guide the RNA-directed DNA methylation pathway (RdDM) to reinforce the chromatin repressive state^17^. Both disruption of DNA methylation patterns and various stresses have been reported to activate TEs transcription and transposition in plants and particularly in maize (*Zea mays*) a fundamental crop for world food supply and model organism for studies of crop genetics, evolution and improvement^18^,^19^,^20^. Maize is an ancient allotetraploid species and has a highly repetitive genome, mainly consisting of TEs (85%): despite the release of its genome sequence in 2009^21^ having increased the amounts of available coding and non-coding transcriptome data, no total transcriptome annotations are available up to now.

In order to improve the predicted gene models and analyze their expression in *Zea mays* under osmotic stress conditions, we produced an extensive set of total RNA-Seq data covering the leaf transcriptome of this crop species after prolonged drought and salt stresses and recovery stages. Osmotic stresses were applied to B73 wild type plants and to the *Required to Maintain Repression 6* mutant (*rmr6*, also known as *rna polymerase d1*-*rpd1-1*, loss of function mutant at the largest subunit of RNA polymerase IV), involved in siRNA biogenesis for the RdDM pathway and required for normal plant development, paramutation, transcriptional repression of certain transposable elements, and transcriptional regulation of specific alleles^22^,^23^. In a recent work^24^ we analyzed the stress-responsive regulation of maize small RNA populations in both B73 wild type and *rmr6* mutant. While 24 nt siRNAs resulted dramatically reduced in *rmr6* mutant, long-term drought stress induces down-regulation of a very small number of siRNA loci in both wt and mutant leaves.

Based on the analysis of our total RNA-Seq data, an improved annotation of the stress-responsive epigenetic-regulated maize transcriptome has been made available. Functional annotation of maize transcriptome has also been improved and, together with the identification of the non-coding portion of the genome, represents an important tool for the whole maize research community. Differential expression analysis revealed hundreds of genes differentially expressed in response to long-term stress application, highlighting also several lncRNAs and transposable elements specifically expressed after osmotic stresses application. Altogether, the reported results represent a valuable tool for further addressing the mechanisms responsible for maize molecular response to abiotic stresses, integrating the regulation of both coding and non-coding portion of the transcriptome.

## Materials and Methods

### Plant materials, stress protocols and tissue collection

The *Zea mays* B73 inbred line and the *rpd1-1* (also known as *rmr6-1*^23^), null mutant, previously introgressed in B73 background by five repeated backcrosses, were used for RNA-Seq analysis. To apply agronomically realistic drought and salinity stresses, the wild type and mutant plants were grown in pots in a greenhouse during spring-summer growing seasons in 2011 and 2012, as described in^24^,^25^ (see also Supplementary Figure S1). Briefly, plants were regularly watered till pot capacity until the V5/V6 developmental stage, when stress treatments were applied: control plants (C) were watered with 75% of disposable water at 0.1dS/m salt concentration; drought-stressed plants (D) with 25% of disposable water at 0.1dS/m salt concentration; salinity-stressed plants (S) with 75% of disposable water at 15dS/m salt concentration; drought plus salinity-stressed plants (D + S) with 25% of disposable water at 15dS/m salt concentration. To mimic the composition of highly saline soils, a complex mixture of salts (Cristal Sea Marinemix^®^) was added to water to reach the defined electrical conductivity values. Treatments were applied daily for ten days. After 10 days of treatment (T0) the youngest wrapped leaf was harvested from a subset of randomly selected plants. The other subset of plants was afterwards watered to pot capacity for seven days to recover from stress and the youngest wrapped leaf was then harvested from each plant (T7). Leaf samples of four-five plants for each combination of genotype-treatment-timepoint were pooled together, flash-frozen in liquid nitrogen and stored at −80 °C. A total of 16 samples (2 genotypes × 4 treatments × 2 time points) were then collected for each of the three biological replicates (R1, R2, R3) produced. All plant materials were sampled between 11.00 AM and 1.00 PM, to avoid as much as possible diurnal variation in gene expression that would obscure the effects of stresses.

### RNA extraction, libraries preparation and sequencing

Total RNA was extracted from frozen tissue using the Spectrum Plant Total RNA Kit (SIGMA) and subjected to On-Column DNase Digestion (SIGMA), according to the manufacturer’s instructions. rRNA depletion was performed with Ribo-Zero™ rRNA Removal Kits (Plant Leaf) from Epicenter-Illumina (Supplementary Figure S1). Libraries for Illumina sequencing were prepared with the TruSeq RNA Library Prep Kit for the first replicate (non-directional sequencing) and with the TruSeq Stranded RNA Library Prep Kit (directional sequencing) for replicates 2 and 3, which were pooled and sequenced together. The Illumina sequencing of the 32 RNA-Seq libraries were performed at the Istituto di Genomica Applicata (Udine, Italy) on an Illumina Hiseq2000 platform with a multiplex level of 4, producing on average 37 million of 50 bp single end reads per library (Supplementary Data S1).

### Total RNA-Seq sequence analysis and genome guided transcriptome assembly

As summarized in Supplementary Figure S1, the sequenced reads were first processed for adapter clipping using Cutadapt 1.2.1^26^ and then trimmed on quality scores and filtered from rRNA contaminant reads with ERNE-FILTER 1.2^27^ using ribosomal RNA sequences retrieved from the “SILVA ribosomal RNA gene database project” (http://www.arb-silva.de/) as contaminant reference. The resulting high quality reads were mapped to the maize B73 reference genome (RefGen ZmB73 Assembly AGPv3 and Zea_mays.AGPv3.20.gtf transcript annotation) with Tophat 2.0.9^28^ using the following modifications from default parameters: maximum intron size, 60,000; minimum intron size, 5; up to three mismatches and gaps allowed. Alignments with MAPQ smaller than 1 and PCR duplicates were filtered out using Samtools^29^. Reads from R2 + R3 sequencing, aligned in strand-specific mode (–library-type fr-firststrand), were used for genome guided transcriptome assembly with Cufflinks 2.2.1 RABT mode^30^,^31^ with –frag-bias-correct, –multi-read-correct and –max-intron-length 60000 options. Based on default parameters, annotation of novel transcripts required the alignment of at least 10 reads in at least one library, and any new isoforms were required to represent at least 10% of the total gene abundance in at least one library. Cufflinks assemblies produced separately starting from the 16 strand-specific libraries were merged using Cuffmerge and the resulting GTF file was edited manually: duplicated reference annotations (i.e. miRNA genes annotated both as GRMZM*** and zma-MIR***) were combined in a single annotation; new annotations shorter than 57 bp (corresponding to the length of the shorter reference transcript) were discarded, while maize ESTs including full-length cDNAs identified by Boerner and McGinnis^32^ from a vast variety of tissues and stages were also collected from GenBank and integrated in the annotation. Based on Cufflinks transfrag class codes, newly identified transcripts were labelled as: i) _j = potential novel isoform at known locus; ii) _O = generic exonic overlap with a reference transcript; iii) _X = transcript overlapping with reference on the opposite strand; iv) TCONS = unknown, intergenic transcript. Generic overlapping, antisense and intergenic transcripts were assigned to new loci named in the same way. Finally, new monoexonic intergenic transcripts spaced by less than 500 bp were fused together in clusters after Blast verification on the merged sequence.

The goodness of the new transcriptome annotation (Zea_mays_new_annotation_final_v5b_sc.gtf; http://www.ncbi.nlm.nih.gov/geo/query/acc.cgi?acc=GSE71046) was verified and validated using RNA-eXpress^33^ and RSEM^34^ softwares.

### Gene ontology annotation and analysis

The full set of 160,488 maize transcripts was analyzed with Blast2GO program^35^ for functional annotation of the gene ontology (GO) terms of newly identified transcripts and implementation of the available GO annotation. Blast2GO functional classification, according to molecular function, biological process and cellular component ontologies has been conducted in two steps: a first BlastX against *Arabidopsis thaliana* proteins (TAIR10; max hits: 1; min coverage between hit and HSP: 30%) and a second alignment of previously not retrieved transcripts against NR database (max hits: 10; min coverage between hit and HSP: 30%; database update of February 2015). Using this approach 77,403 transcripts were functionally annotated (Supplementary Data S2). The GO terms were exported to WEGO GO plotting tool^36^, categorized using level 2 of the GO lineage and compared with the maize GO annotation available at EnsemblPlants/Biomart database on February 2015 (Supplementary Figure S2). Finally, Ontologizer^37^ software was used for GO-enrichment analysis, using the term-for-term approach for overrepresentation statistical analysis, with a classic Bonferroni correction for multiple testing. GO enrichments were summarized using REVIGO^38^ and represented as scatterplots in which the enriched terms remaining after the redundancy reduction are represented in a two dimensional space derived by applying multidimensional scaling to a matrix of the GO terms’ semantic similarities. The guiding principle is that semantically similar GO terms should remain close together in the plot. Bubble colour indicates the p-value (legend in upper right-hand corner); size indicates the frequency of the GO term in the underlying GOA database (bubbles of more general terms are larger)^38^.

### lncRNA identification

A specific pipeline was developed in order to predict the potential long non-coding RNAs in the newly annotated transcriptome. The pipeline was developed on the basis of the criteria currently used to distinguish the long non-coding from coding transcripts: i) a length > = 200 bp; ii) the presence of an Open Reading Frame <120 amino acids; iii) when an ORF is present, the predicted protein must not match any protein in public databases^39^. In this way, 36,151 transcripts were identified as potential long non-coding RNA (pot-lncRNAs). To help in the classification of these transcripts, each long non-coding RNA was compared with the publicly available sequences of smallRNA precursors^40^ and TE-elements of maize (http://maizetedb.org/~maize/) and with the database of smallRNA identified through smallRNA-Seq on the samples used for the RNA-Seq experiment^24^ using blastn with the following parameters: e-value < 0.01, 100% of identity and word-size of 16. 22,764 transcripts had a match in the abovementioned databases, thus suggesting that they might be precursors of smallRNAs (21,624) or expressed from transposable elements (1,140). The remaining 13,387 transcripts were annotated as “truly” long non-coding RNAs (Supplementary Data S5). Comparisons with lncRNAs identified in^41^ were made with the BEDTools function intersectBed^42^ after liftover of transcript genomic coordinates using the Assembly Converter at EnsemblPlants (http://plants.ensembl.org/tools.html). Retrieval of mitochondrion (Mt) and chloroplast (Pt) loci coordinates was unsuccessful, so they were excluded from the comparison.

We defined as long non-coding Natural Antisense (lncNAT) transcripts those lncRNAs transcribed from the opposite strand of an annotated gene with at least a 10-nt overlapping sequence (Supplementary Data S5). Intersections of genomic coordinates between lncRNAs and coding transcripts were performed with the intersectBed tool. To determine whether or not the remaining lncRNAs, classified as lincRNAs, could regulate the expression of protein coding genes as long molecules, they were tested for homology to CDS sequences using Blastn (more than 95% identity and minimum overlap of 100 bp) against the nucleotide sequence of the 124,337 coding transcripts.

### Differential expression analysis

Pair-wise differential expression analyses at gene and transcript level were obtained with Cuffdiff^31^ selecting the following options: –multi-read-correct, –compatible-hits-norm, –dispersion-method per-condition and –library-norm-method quartile options were selected.

Pairwise differential expression analyses to identify stress modulated genes were performed combining the sequenced samples in different groups, considering the different stress application as replicates: a) B73-Stress_set (B73:C_T0 vs B73:D_T0 + B73:S_T0 + B73:D + S_T0) and b) *rmr6*-Stress_set (*rmr6*:C_T0 vs *rmr6*:D_T0 + *rmr6*:S_T0 + *rmr6*:D + S_T0). A further analysis was conducted to evaluate the mutation effect in control conditions: c) Mutant_set (B73:C_T0 vs *rmr6*:C_T0). Finally, two additional pairwise differential expression analyses were conducted to determine the effect of the recovery seven days after the removal of the stress: d) B73-Recovery_set (B73:C_T7 vs B73:D_T7 + B73:S_T7 + B73:D + S_T7), and e) *rmr6*-Recovery_set (*rmr6*:C_T7 vs *rmr6*:D_T7 + *rmr6*:S_T7 + *rmr6*:D + S_T7).

Differential expression estimations were carried out at both gene and transcript level: genes and transcripts with log2 fold change ratio ≥|1| and FDR- adjusted p value ≤ 0.05 were considered as statistically differentially expressed (DE; Supplementary Data S7 and S8), while features with test status = NOTEST or LOWDATA in all three expression analyses (roughly corresponding to RPKM <1 in all the conditions; Reads Per Kilobase Of Exon Per Million Reads Mapped) were considered as not expressed. In addition, we excluded from downstream analysis 62 genes located in a region of 30 Mb surrounding the *RMR6-1* locus, resulting differentially expressed (30 up- and 32 down-regulated) in the Mutant_set analysis: their misregulation could be due to the sequence polymorphisms (increasing gene expression or impairing read mapping) between the introgressed *rpd1/rmr6* mutant and the B73^22^.

### Clustering of differentially expressed genes (DEGs)

Expression profiles of DEGs under the three different osmotic stresses and after the recovery stage in both B73 and *rmr6* mutant were achieved using the Short Time-series Expression Miner (STEM) software^43^. This algorithm uses a unique method to cluster time series gene expression data and it also allows to directly compare the expression profiles obtained from two different experimental conditions, permitting automatic identification of statistically significant sets of genes which are (or are not) co-expressed under the two experimental conditions (B73 and *rmr6* mutant in this study). For comparison of expression profiles under different osmotic stresses, all significantly DE genes at T0 in B73 and/or *rmr6* were clustered into 18 distinct expression patterns (Supplementary Figure S3), starting from their RPKM values in each single growth condition. For dynamic expression analysis after the stress application and the recovery stage, the T7/T0 ratio for each growth condition was plotted for all DE genes in at least one timepoint (Supplementary Figure S4). RPKM values were quantified in each sample (growth condition, timepoint and genotype) with Cuffquant and normalized with Cuffnorm^31^.

### Transposable elements annotation in between maize transcripts

Given the high content in transposable elements (TEs) of the maize genome and the redundancy of the RefGen ZmB73 RepeatMasked Assembly AGPv3, transcript sequences related to transposable elements were identified and classified using BLAST Best Hits. All 160,488 transcript models were BLASTed against the Maize TE database containing 1,526 full-length sequences of curated, non-redundant maize TEs (http://maizetedb.org/~maize/). The bit score and coverage percentage of the alignment were scored to identify TE-related transcripts with stringent criteria and classify them in two subgroups: 9,766 high-confident-TEs (HC-TEs: with Bit-score >500 and coverage >50%) and 9,013 putative/relics-TEs (PR-TEs: with Bit-score >250 or coverage >30%). Each TE-related transcript was then associated with its specific TE-family and superfamily to analyze the preferential transcription of specific TE-classes in mutant samples.

### Novel transcripts confirmation by sequencing of RT-PCR amplicons and Q-PCR expression validation

To confirm the existence of the described newly identified splicing variants and intergenic transcripts, specific primers (Supplementary Table S4) were designed and used in RT-PCR amplification experiments. cDNA synthesis was performed with the SuperScript III reverse transcriptase kit (Invitrogen), according to the manufacturer’s instructions. One microgram of total RNA (extracted as previously described) was used as a template together with 1 μl oligo (dT) 12–18 (0.5 μg/μl–Invitrogen). Each cDNA was then diluted 1:10 and 1 μl was used for PCR amplification of specific transcripts in a volume of 25 μl. PCR reactions were checked on 1.2% agarose gels stained with Sybr-Safe (Invitrogen). Single amplified fragments were gel purified using the QIAquick Gel Extraction Kit (Qiagen) and directly Sanger sequenced on both strands at BMR Genomics (http://www.bmr-genomics.it/seq_index.html). Sequences were edited and aligned against the reference transcripts using Geneious.

Quantitative Real-Time PCR expression analysis was performed using an ABI 7500 Real-Time PCR System (Applied Biosystems) and the POWER SYBR^®^ GREEN PCR Master Mix (Applied Biosystems) following the manufacturer’s guidelines. Real-time conditions were: 2 min at 50 ˚C, 10 min at 95 ˚C, 40 cycles of: 15 s at 95 ˚C and 1 min at 60 ˚C. For each reaction, the product melting curves were determined by heating from 60 to 95 ˚C at 0.2 ˚C/s. For all transcripts, a single product was identified. Three replicates were carried out for each primer combination and a relative quantification of gene expression (normalized to *GAPC2* transcript quantities) was performed. Primer sequences are reported in Supplementary Table S4.

## Results and Discussion

### RNA Seq profiling of leaf total transcriptome in B73 and *rmr6-1* mutant

To define the leaf-transcriptome of maize B73 wild type and PolIV mutant subjected to agronomically realistic drought and salinity stresses, a total-RNA-Seq approach was applied (Supplementary Figure S1). Plants belonging to B73 inbred line and *Required to Maintain Repression 6* mutant (*rmr6/rpd1-1*, involved in siRNA biogenesis and in the RNA-directed DNA methylation pathway^23^, introgressed into the B73 background), were grown until 6^th^ leaf stage and then subjected for ten days (T0) to control growth condition (C), drought stress (D), salt stress (S) and combined stress (D + S). After the stress application, the stressful conditions were removed and the plants grown for 7 days in control conditions (T7). The stress experiment was repeated three times; total RNA was isolated and subjected to rRNA removal (see Materials and methods) prior to sequencing on a HiSeq2000 Illumina Instrument. After quality trimming and filtering of contaminant rRNA reads, 584,396,980 and 622,948,010 high quality reads were aligned to the maize B73 reference genome (RefGen ZmB73 Assembly AGPv3) for B73 and *rmr6*, respectively (Table 1), with a mean mapping rate of about 98% (Supplementary Data S1). Reads mapping with MAPQ smaller than 1 (corresponding to more than 10 alignments to the genome) and PCR duplicates were filtered out, and the number of filtered reads and alignments are reported in Table 1 and Supplementary Data S1.

### Reference guided transcriptome assembly of maize leaf

**Figure 1:**
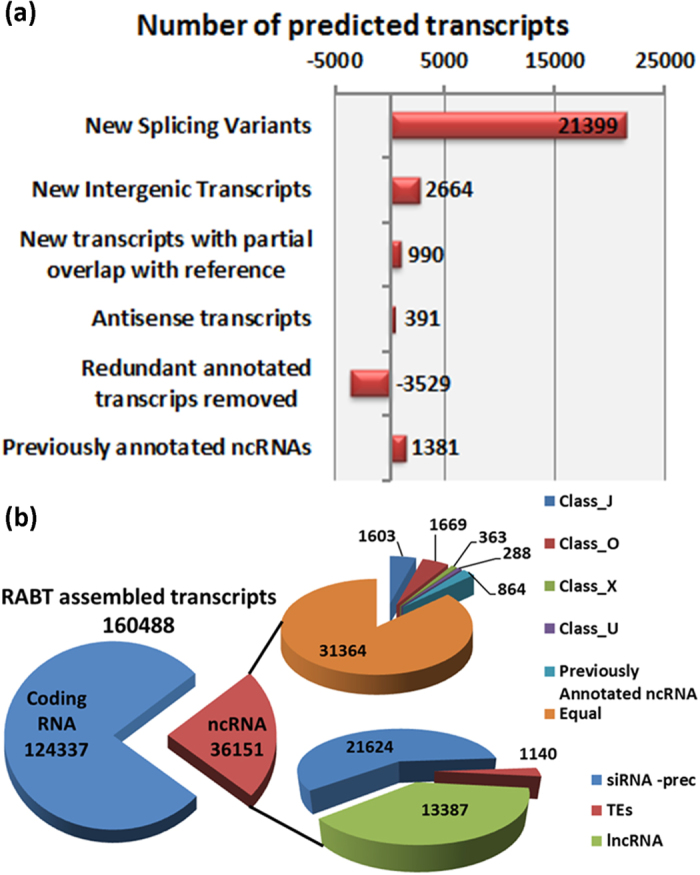
Identification and classification of novel gene models and non-coding RNAs. (**a**) RABT identified transcripts were classified, based on Cufflinks transfrag class codes, in: i) newly identified isoform at known locus (J); ii) unknown, intergenic transcript (U); iii) generic exonic overlap with a reference transcript (O) and iv) transcript overlapping with reference on the opposite strand (X). (**b**) Application of a specific pipeline for lncRNAs identification and classification allowed the identification of 36,151 ncRNAs that were further classified based on their annotation class or their putative homology. ncRNAs without any si/shRNA or TE homology were labeled as truly lncRNAs.

In order to assemble the maize total leaf transcriptome, the high quality filtered reads deriving from the strand-specific sequencing were used for reference guided transcriptome assembly (Supplementary Figure S1) with Cufflinks^31^. In order to avoid false positives, only the transcripts expressed at least in one condition and with a minimum length of 57 bp were retained. Our new “Reference Annotation Based Transcript” (RABT) allowed the identification of 114,382 loci corresponding to 160,488 transcripts (137,192 transcripts at 110,451 loci were present in the reference Zea_mays.AGPv3.20.gtf transcript annotation). The re-annotation identified 21,399 new isoforms (Class J), 2,664 new intergenic transcripts (Class U), 990 transcripts that partially overlapped annotated transcripts (Class O) and 391 antisense transcripts mapping with opposite orientation in respect to reference transcripts (Class X). We could also resolve 850 previously annotated loci (corresponding to 3,529 transcripts) representing redundant annotations. Finally, we included in the final version of the re-annotated maize leaf transcriptome 1,381 nonredundant, non-coding transcripts identified in the work of Boerner and McGinnis, 2012 (Fig. 1a).

The goodness of the new transcriptome annotation was verified using RNA-eXpress software^33^: starting from the same libraries and applying the same filters, 70% of the 2,664 new transcripts were confirmed, with a mean overlap of 80%. The analysis of the transcripts not confirmed by this approach showed that they are localized in wide repeated genomic regions, rich in *rmr6-*derived reads, in which the two algorithms annotated transcripts with not-overlapping coordinates. Annotation was further validated using the RSEM package^34^, re-mapping the cleaned reads against the reconstructed transcriptome and estimating isoforms expression. With this independent assay, 99% of Class X and Class U transcripts passed the expression arbitrary threshold 1 RPKM (Reads Per Kilobase per Million mapped reads) in at least one condition. The percentage decreased slightly for Class O (79%) and Class J (66%) but it was twice higher than those of reference transcripts (35%).

### Gene Ontology (GO) assignment

It is reasonable to question what proportion of the novel maize genes is likely to have a biological function. Therefore, to gain further insight into the newly identified transcripts and to improve the maize transcriptome functional annotation in terms of cellular components, biological processes and molecular functions, we used the Blast2GO suite^35^: the full set of 160,488 transcripts were first aligned against *Arabidopsis thaliana* proteins to assign a GO term; transcripts without a match were then re-aligned against NR protein database (see Materials and methods for detailed description), successfully assigning GO terms to 77,403 transcripts with an average of 7.2 terms per transcript (Supplementary Data S2).

We traced 46,817 transcripts to biological process terms (52.62% of terms), 66,057 transcripts to cellular component terms (34.91% of terms), while 50,389 transcripts were linked to molecular function terms (12.47% of terms). The WEGO annotation plotting tool^36^ was then used to compare the new GO annotation with those available in the EnsemblPlants/Biomart database (38,222 transcripts with GO terms assigned). As shown in Supplementary Figure S2, overall distribution of transcripts annotated in molecular function subcategories is very similar in the two annotations, while biological process and cellular component categories are slightly over-represented in the new GO annotation.

**Figure 2:**
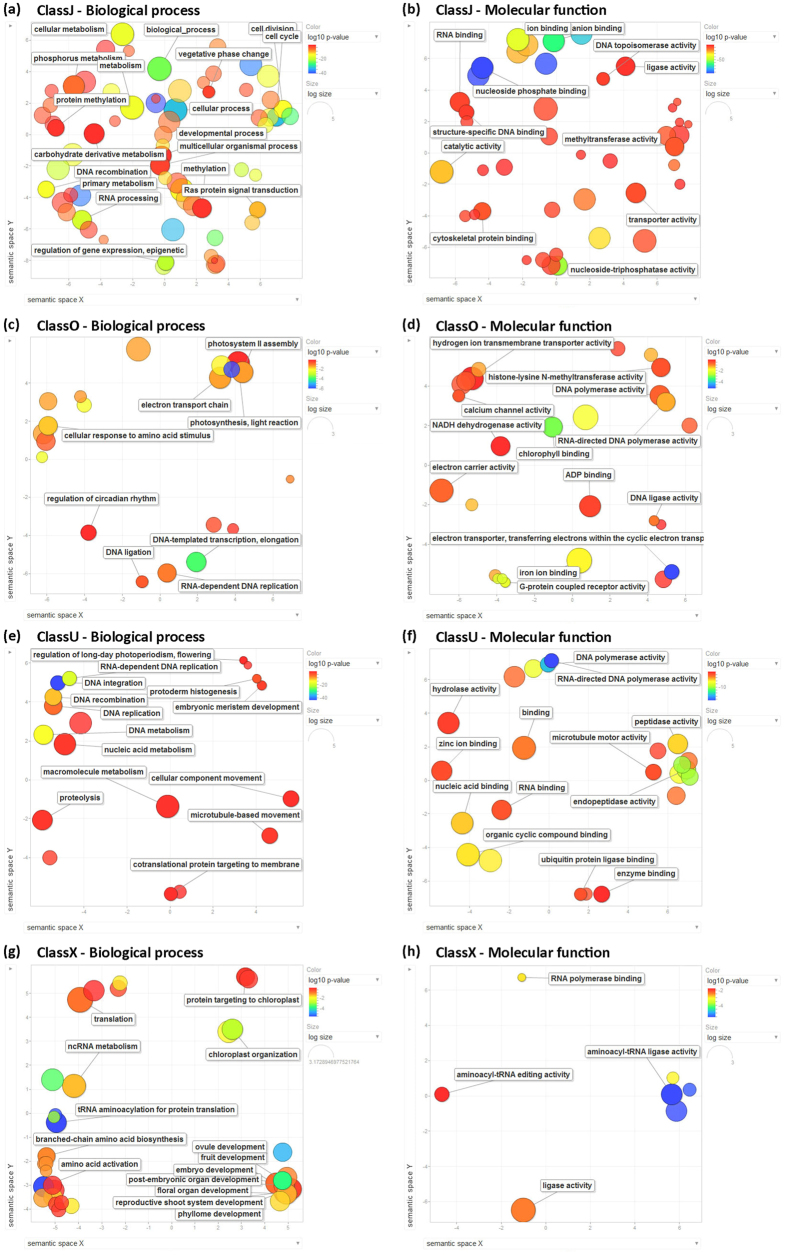
Results of REViGO semantic analysis of GO biological process and molecular function terms enriched in newly annotated transcript classes. Functional enrichment of GO-terms was independently analyzed in the newly annotated transcript classes with Ontologizer software^37^ and summarized using REVIGO^38^. For each class, enriched terms remaining after the redundancy reduction are represented as scatterplots in a two dimensional space which summarize GO terms’ semantic similarities. The guiding principle is that semantically similar GO terms should remain close together in the plot, but the semantic space units have no intrinsic meaning. Bubble color indicates the p-value for the false discovery rates; circle size indicates the frequency of the GO term in the underlying GO database (bubbles of more general terms are larger; http://revigo.irb.hr/).

**Figure 3:**
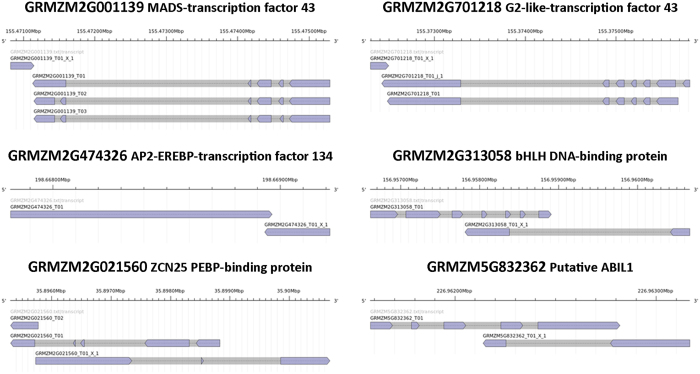
Schematic representation of newly identified antisense transcripts and their paired sense transcripts. Indicative but not exhaustive representation of *de-novo* identified antisense transcripts mapping on the opposite strand of genes important for plant development. Gene structures were obtained with the AnnotationSketch online tool (http://genometools.org/cgi-bin/annotationsketch_demo.cgi) using the maize assembled transcriptome. Full list of antisense X locus/reference transcripts pairs is reported in Supplementary Data S4.

Specific GO terms were assigned to more than 83% (17,942 out of 21,399) of new isoforms and to 40% (392/990) of ClassO transcripts, while only 9% of intergenic and 4% of antisense transcripts were functionally annotated, indicating a low protein coding potential of the latter two classes. Functional enrichment of GO-terms analysis, performed with Ontologizer software^37^ and summarized using REVIGO^38^ (Fig. 2 and Supplementary Data S3), revealed that about 560 GO terms are enriched in the newly annotated isoforms (Class J), including mitosis/cell cycle-, gene expression regulation-, RNA-mediated gene silencing-, chromosome organization-, protein modification-, phosphorous metabolism-, DNA binding and ion binding-related terms and many others (Fig. 2a,b). Class O transcripts are enriched in GO annotations linked to photosynthetic, electron transport chain, ion transport and RNA-dependent DNA replication (Fig. 2c,d). This GO category is also enriched within the few Class U annotated sequences, together with DNA recombination/integration, peptidase activity and protoderm and meristem differentiation terms (Fig. 2e,f). Finally, antisense transcripts (Class X) are enriched in ncRNA metabolism, plastid differentiation and plant embryonic and post-embryonic development-associated GOs (Fig. 2g,h). On the other side, the corresponding sense mRNAs are enriched in DNA binding and transcription factor GO terms (Supplementary Data S3), and several *de-novo* identified antisense transcripts map on the opposite strand of genes important for plant development. Besides transcription factors of several different families, antisense transcripts were identified at the locus encoding for *ZCN25*, a phosphatidylethanolamine-binding protein (PEBP) family protein homologous to Arabidopsis and rice Flowering Locus T involved in flowering regulation^44^, at maize *ABIL2* orthologous gene, involved in regulation of actin and microtubule organization (Fig. 3) and many others (Supplementary Data S4).

### Identification and characterization of maize lncRNAs

To predict potential maize long non-coding RNAs, the comprehensive set of transcripts was then analyzed with a specific pipeline developed on the basis of the criteria currently used to distinguish the lncRNAs from coding or other short non-coding transcripts: i) length > = 200 bp; ii) presence of an Open Reading Frame <120 amino acids; iii) when an ORF is present, the predicted protein must not match any protein in public databases^39^. In this way, 36,151 transcripts were identified as potential long non-coding RNA (pot-lncRNA). 86.7% of these pot-lncRNAs were present in the Zea_mays.AGPv3.20.gtf transcript annotation, while about 3,900 were *de-novo* annotated (Table 2). The percentages of pot-lncRNAs in the two subsets of genes is quite different (31,364 out of 133,663, 23.46% of reference annotations against 3,923 out of 25,444, 15.42% of RABT transcripts) and it is also worth noting that 72% of pot-lncRNAs identified within the Ensembl annotation are expressed below 1 RPKM in our leaf samples (see Materials and methods for expression quantification and expression filtering settings) compared to 19% of low expression RABT ones.

To help in the classification of these transcripts, each potential long non-coding RNA was compared with the publicly available sequences of shRNA, siRNA, miRNA, rRNA and TE-elements of maize and with the database of smallRNA loci identified through smallRNA-Seq on the same samples^24^. This comparison matched 22,764 (63%) transcripts in the abovementioned databases thus suggesting that they might be precursors of smallRNAs or expressed from transposable elements. The remaining 13,387 transcripts were annotated as “truly” long non-coding RNAs (Fig. 1b, Table 2 and Supplementary Data S5). These include 11,815 Ensembl maize annotations and 1,223 sequences from *de novo* transcripts assembly.

We included in the transcriptome annotation, and consequently in our lncRNAs identification analysis, 1,381 no-redundant, non-coding transcripts identified in 2012 by Boerner and McGinnis^32^ starting from a set of 24,467 full-length cDNAs. Our pipeline included more than 60% of these sequences in pot-lncRNAs, while the remaining 40% resulted as coding transcripts.

A more recent paper reported the identification of maize lncRNAs from the *de novo* transcript assembly of 30 different RNA-Seq experiments^41^, starting from Working Gene Set transcripts (WGS) and the maize AGPv2 genome assembly. The authors identified 20,163 putative lncRNAs (12,648 isoforms from the WGS and 7,515 isoforms from the *de novo* transcript assemblies) that were subdivided in 18,459 pre-lncRNAs (potential precursors of siRNA and other small RNAs) and 1,704 high-confidence lncRNAs (HC-lncRNAs). The smaller number of pre- and HC-lncRNAs reported by Li *et al.* within the WGS transcripts, compared to our pot- and truly-lncRNAs, is the consequence of an EST-based expression filter applied to remove about 21,600 WGS isoforms without expression evidence. As a consequence, the number of high confidence lncRNAs is eight-fold lower than ours. On average, 95% of lncRNAs reported by Li *et al.* were confirmed and included in our annotation, which also includes 16,000 additional lncRNA candidates that were previously excluded, most certainly due to the expression filter applied by the authors.

Recently, the expression levels of 4,789 of these 16,000 lncRNA were analyzed in a comprehensive and systematic RNA-Seq transcriptome profiling of 79 distinct maize tissues and organs (only 63,272 transcripts were included in this study because the authors focused their analysis on a single transcript, the longest one, to represent each gene)^45^,^46^. The screening of this expression atlas revealed that 3,481 (72.6%) of them are expressed (FPKM >1) in at least one analyzed tissues or developmental stages (Supplementary Data S6). Similarly, the screening of RNA-Seq data included in the qTeller tool (http://www.qteller.com/) indicated that 3,621 of 4,468 loci (81%) producing at least one non-coding transcript not included by Li *et al.*, passed the 1 FPKM expression threshold in at least one tissue (Supplementary Data S6), confirming the actual transcription of these ncRNA during maize growth and development.

Recent studies on mammals and plants classified lncRNAs as originating from the natural antisense transcription (NAT lncRNAs) of known loci or from the transcription of intergenic regions (long intergenic ncRNAs: lincRNAs). The former class is of particular interest because these lncRNAs could act as positive or negative regulators of the sense coding transcript annotated at the same locus, while the latter may be part of epigenetic regulatory pathways such as the smallRNAs, DNA methylation and histone modifications pathways. Based on this classification, the 13,387 truly lncRNAs could be further subdivided in 3,012 NAT lncRNAs and 10,375 lincRNAs while within the *de novo* annotated transcripts we identified 234 NAT lncRNAs and 989 lincRNAs (Supplementary Data S5).

The length of lncNATs varies from 201 to 3,780 nt with an average of 535 nt. The majority of NAT pairs (2,157 pairs, corresponding to 70%) consisted of the identified lncNAT and a protein coding mRNA but we also found 750 NAT pairs made up of two complementary lncNATs and 330 pairs composed of paired lncNAT and siRNA precursors (Supplementary Data S5).

The average length of lincRNA is 465 nt, with an interval ranging between 201 and 4,179 nt. They could either regulate the expression of protein coding genes at epigenetic, transcriptional and post-transcriptional level working as a molecular guide or scaffold for chromatin modifying enzymes or acting as decoys to DNA-binding proteins, such as transcription factors, chromatin modifying proteins but also microRNAs. In the first case the lncRNAs mediate the recruitment of particular protein complexes to homology containing target loci, while in the second one the sequence homology to the target gene will result in the binding of the specific effectors preventing their interaction with the target gene^14^,^47^. To identify the putative targets of maize lincRNAs, they were then tested for similarity with protein coding CDS using BLAST. Of 10,375 lincRNAs, 575 present a perfect match (100% identity, minimum overlap 100 bp) with coding CDSs (a further 1,780 lincRNAs display a near exact match, with more than 95% of identity, see Supplementary Data S5). Putative transcription factors but also metabolism and development-related proteins are enriched within the putative targets of lincRNAs.

### Differential expression in response to osmotic stresses in wt and *rmr6* mutant

**Figure 4:**
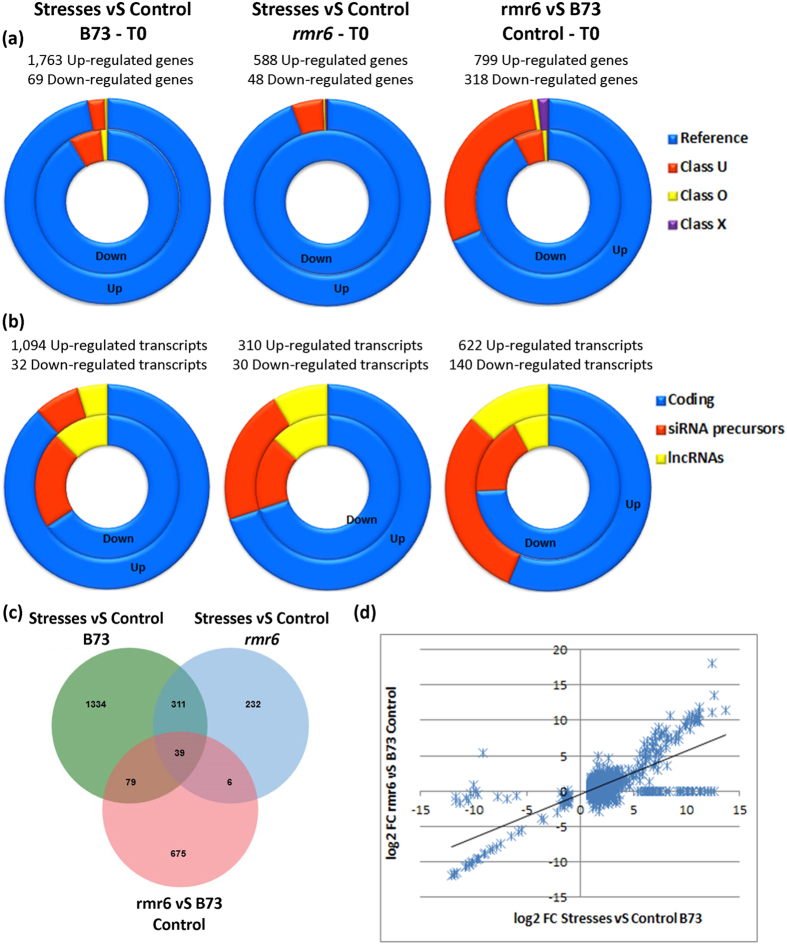
Summary and comparison between genes and transcripts differentially expressed at T0. Summary of genes (**a**) and transcripts (**b**) differentially expressed in each pairwise comparison divided by their annotation class (**a**), or based on the coding/no coding classification (**b**). In the ring graphs, the inner ring depicts the down-regulated genes and the outer the up-regulated ones. **c**) Venn diagram representing the up-regulated transcripts common between the three differential expression tests. (**d**) Correlation between log2 FC obtained in B73 stressed test and in *rmr6* control test for the subset of transcripts differentially expressed in B73 in response to osmotic stresses.

To gain further insights into stress- and epigenetic- mediated transcription of the maize genome coding and non-coding portions, we analyzed differential expression using Cuffdiff^31^. To obtain a global view of stress and epigenetic components behind transcriptional changes and their potential relationship, we performed three independent pairwise comparisons joining the sequenced samples in different combinations: i) B73-Stress_set (B73:C_T0 vs B73:D_T0 + B73:S_T0 + B73:D + S_T0), ii) *rmr6*-Stress_set (*rmr6*:C_T0 vs *rmr6*:D_T0 + *rmr6*:S_T0 + *rmr6*:D + S_T0) and iii) Mutant_set (B73:C_T0 vs *rmr6*:C_T0). Since lncRNAs and TEs were annotated at transcript level, differential expression estimations were carried out at both gene and isoform level. Genes or transcripts with log2 fold change ratio ≥|1| and FDR- adjusted p value ≤ 0.05 were considered as statistically differentially expressed and because of multiple isoforms at a single gene can be differentially modulated, the two approaches gave slightly different results in terms of number of features modulated (Fig. 4a,b and Supplementary Tables S1 and S2; see also Supplementary Data S7 for results of differential expression tests).

The three independent expression analyses gave very different results in terms of differentially expressed genes (DEGs), considering both total number of hits and their distribution between annotation classes and coding potential (Fig. 4a,b; Supplementary Tables S1 and S2).

Osmotic stress applications in wild type plants caused the miss-regulation of the highest number of genes and transcripts (mainly their up-regulation), and more than 96% of them are represented by reference annotated genes, indicating a high impact of stresses on gene regulation. On the contrary, the same stresses applied to the *rmr6* mutant seem to have a very low effect on transcription, with about one third of genes miss-regulated compared to wild type plants. In the mutant the majority of DE genes consist of reference annotations, but a significant percentage is also represented by newly annotated intergenic loci. ClassU intergenic loci represent instead about the 30% of genes differentially expressed between the two genotypes in control growth condition (Fig. 4a; Supplementary Table S1). The high impact of the *rmr6* mutation on transcriptional regulation of newly annotated genes (but also of reference transcripts), even in non-stress condition, must be taken into account to explain the differences in stress responses between the genotypes.

As predictable, GO analyses reported significant enrichment for stress-response terms in both B73 and *rmr6* stress-upregulated genes, with 541 (30%) and 152 (25%) of genes falling in this ontology, respectively. It is worth noting that the stress-response ontology term is significantly enriched also in between the genes upregulated in *rmr6* vs B73 in control growth conditions: 105 of the 799 upregulated genes (13%) fall in this ontology. Forty-four of these genes were also up-regulated in B73 plants subjected to stress treatments (Fig. 4c and Supplementary Table S3).

These observations point to a stress-like transcriptional response caused by the *rmr6* mutation, which appears even more evident plotting the log2FC ratio of all transcripts differentially expressed in B73 stressed plants against their respective value in *rmr6* vs B73 in control condition. The expression variations in the two experiments are indeed positively correlated (Pearson correlation = 0.741, P < 0.01; Fig. 4d).

All together, these results explain the reduced number of DEGs following stress application in the *rmr6* mutant: being already expressed at higher level in control condition, they are not further upregulated by the stress treatment (see expression values in Supplementary Data S8).

This “stress-like” effect caused by the loss of PolIV activity also resulted in the transcriptional activation of a huge number of genomic loci previously not annotated in the genome. The forty percent of the 230 Class U genes overexpressed in *rmr6* compared to B73 presents TE-related transcripts, mainly of Copia and Gypsy LTR superfamilies. Considering also the reference annotations, about 20% of the transcripts upregulated in mutant background were represented by TE-related genes, while lower percentages were induced by stresses application (Table 3). Gypsy, Copia and unclassified LTR families were over-represented in all three sets of upregulated transcripts, with also an interesting percentage of transcripts ascribable to the DTA/hAT family of classII DNA transposon induced by stresses application in B73.

Besides TEs, hundreds of non-coding transcripts are over-expressed in *rmr6* mutant (44% and 34% of up-regulated transcripts in control and stress condition, respectively), compared to the 12% observed in B73 under osmotic stresses. Between differentially expressed ncRNAs, siRNA precursors are more represented than truly lncRNAs in *rmr6* genotype, while the two classes are almost equally distributed within the B73 stressed-set (Fig. 4b; Supplementary Table S2).

### Comparison of differentially expressed genes under different osmotic stresses

**Figure 5:**
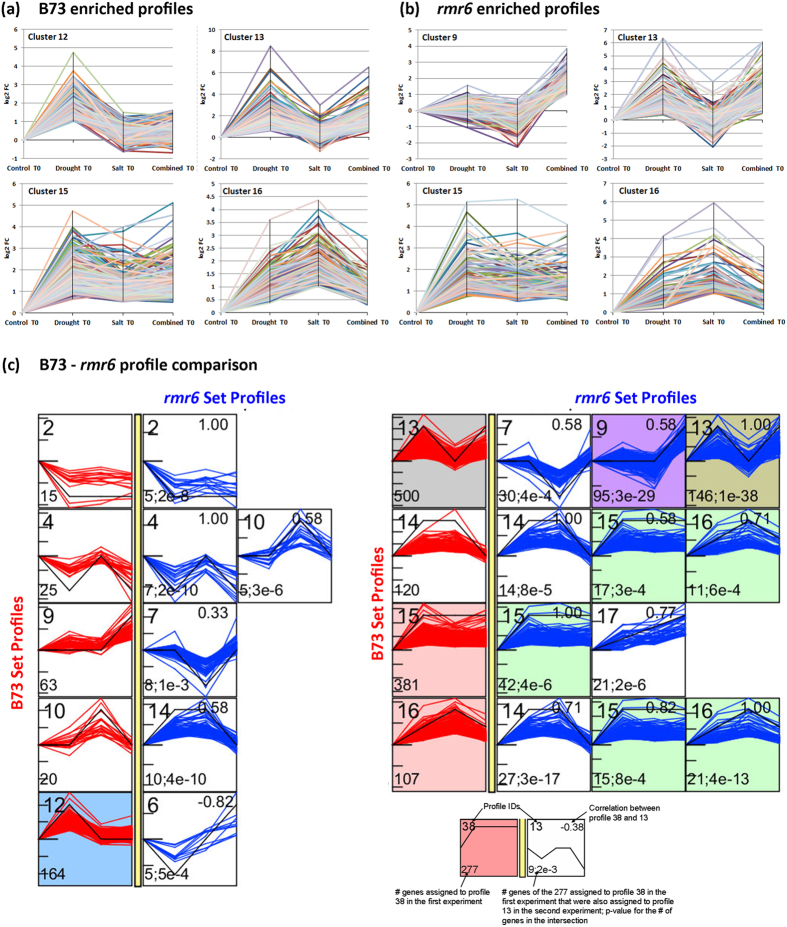
Comparison of DEG profiles under different osmotic stresses. STEM clustering^43^ was used to identify common expression profiles of DEGs under the three different stress conditions. Statistically enriched expression profiles are reported for B73 (**a**) and *rmr6* (**b**); see Supplementary Figure S3 for the whole set of identified profiles and clusters. STEM software was also used to compare the gene sets associated to each expression profiles in the two genotypes to identify commonly or differentially modulated genes (**c**). The legend for the comparison interface was taken from the STEM manual (http://www.sb.cs.cmu.edu/stem/): a profile to the right of the yellow bar is from the *rmr6* experiment, and has a significant intersection (in terms of the genes assigned to them) with the profile to the immediate left of the yellow bar in its row (derived from B73 experiment).

To survey the maize transcriptional response under different stress conditions, we employed STEM (Short Time-series Expression Miner) software package^43^, classifying all DEGs according to their expression profiles after the application of three different stresses in B73 and *rmr6*. The 2,113 significantly DE genes at T0 in B73 and/or *rmr6* were clustered into 18 distinct expression bases on the log2 FC calculated for each single stress compared to the unstressed control (Supplementary Figure S3). Clusters were ordered according to the number of co-expressed genes (reported on the bottom-left corner) and four significantly enriched expression profiles (that have a statistically significant number of genes assigned, compared to the number of genes expected based on the permutation test) were identified in B73 and *rmr6*. Significant profiles are represented by different background colours in Supplementary Figure S3 and are reported in Fig. 5a,b. Profiles 13 (strong induction by drought and combined stress, not affected by salt stress), 15 (up-regulation in all the three stresses) and 16 (strongest effect of salt stress) resulted significantly enriched in genes (compared to the number of expected genes) in both lines (Fig. 5a,b). Conversely, profiles 12 (up-regulation following only drought stress) and 9 (up-regulation only in combined stress), resulted specifically enriched exclusively in B73 or *rmr6*, respectively. All profiles with genes negatively affected by the stress application (i.e. 1, 2, and 4) resulted not significantly enriched given the initial low number of stress down-regulated genes (Supplementary Figure S3).

Comparison and intersection of gene expression profiles of B73 and *rmr6* revealed that many long-term stress-responsive genes are co-expressed (or similarly expressed) in both genotypes, and also confirmed the “stress-like” effect caused by the loss of PolIV activity (Fig. 5c). For example, 125 genes assigned to profile 13 (up-regulated by drought and combined stresses) in B73 were instead included in profiles 7 (no effect of drought and combined stresses, downregulation following salt stress) and 9 (induced by only the combined stress) in *rmr6*.

### Dynamic expression profiles of stress response and recovery stage

To understand the transcriptional changes associated to the recovery from the osmotic stresses we performed two additional differential expression analyses on B73 and *rmr6* leaves collected after 7 days of recovery; as was done previously, the pairwise comparisons were named: i) B73-Recovery_set (B73:C_T7 vs B73:D_T7 + B73:S_T7 + B73:D + S_T7), and ii) *rmr6*-Recovery_set (*rmr6*:C_T7 vs *rmr6*:D_T7 + *rmr6*:S_T7 + *rmr6*:D + S_T7).

**Figure 6:**
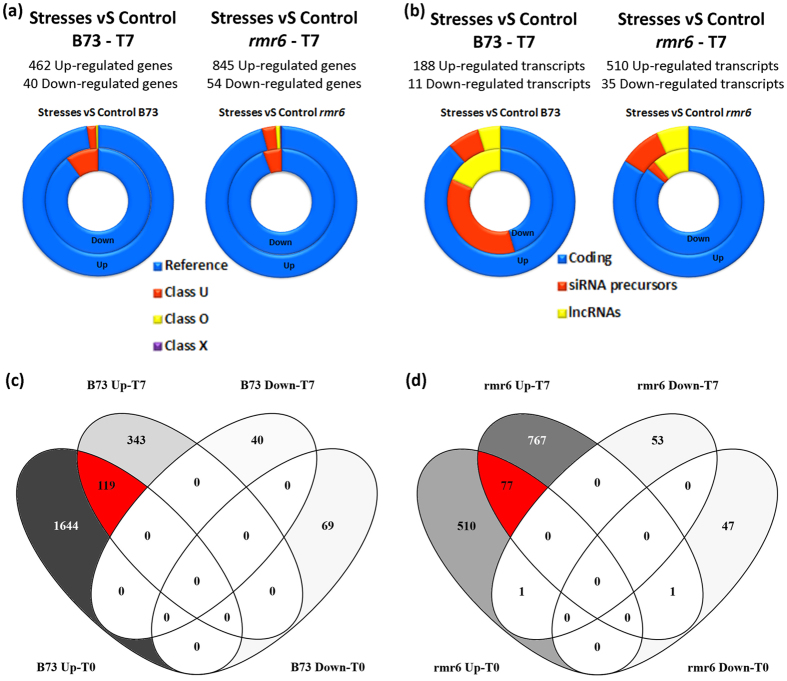
Summary and comparison between genes and transcripts differentially expressed at T7. Summary of genes (**a**) and transcripts (**b**) differentially expressed in each pairwise comparison divided by their annotation class (**a**), or based on the coding/no coding classification (**b**). In the ring graphs, the inner ring depicts the down-regulated genes and the outer the up-regulated ones. (**c,d**) Venn diagrams produced with VENNY (http://bioinfogp.cnb.csic.es/tools/venny/) comparing the DE genes upon stress and recovery period.

Even after the recovery, B73 and *rmr6* display a very different number of DEGs, but opposite to what observed at T0, mutant plants recovered from the stress showed the misregulation (almost exclusively the up-regulation compared to the not-stressed T7 samples) of an higher number of genes and transcripts than B73 plants (Fig. 6a,b; Supplementary Tables S1 and S2): 845 genes (510 transcripts) were up-regulated in *rmr6* compared to the 462 (188) of B73. DEGs are mainly represented by protein-coding, reference annotated genes, although several lncRNA and siRNAs were also DE (Fig. 6b). In both genotypes, plants that experienced osmotic stresses maintain higher expression level of many stress-responsive genes that were previously found up-regulated at T0 (Fig. 6c,d). It is worth noting that 119 of the 1,763 initially up-regulated genes (6.7%) were still over-expressed after the recovery in B73, compared to the 13% (77 of 582) in *rmr6*.

**Figure 7:**
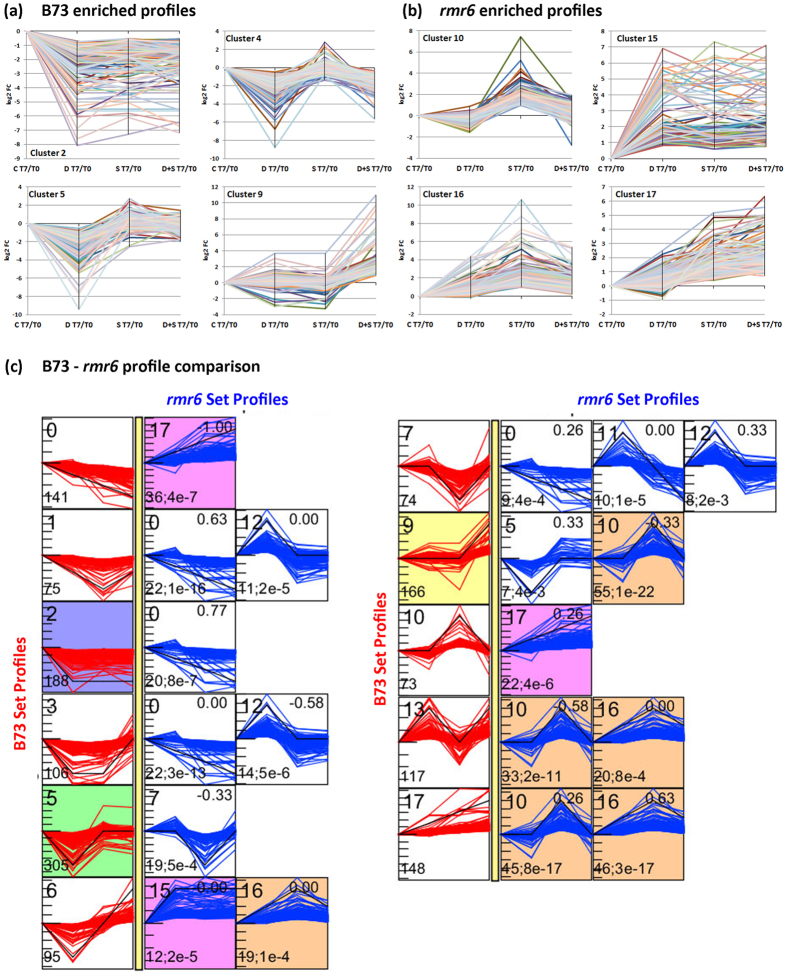
Comparison of DEG profiles under stress response and recovery stage. STEM clustering^43^ was used also to analyze the expression dynamics DEGs under the three different stress conditions and the recovery stage, plotting the to the log2 T7/T0 expression ratio of each DE gene for the three analyzed stresses. Statistically enriched expression profiles are reported for B73 (**a**) and *rmr6* (**b**); see Supplementary Figure S4 for the whole set of identified profiles and clusters. Comparison of the gene sets associated to each expression profiles in the two genotypes revealed completely different stress-recovery dynamics in the two genotypes (**c**).

To analyze the expression dynamics of stress recovery in B73 and *rmr6* we clusterized and compared the DEG expression profiles after the application and removal of the three different stresses. STEM software was used to identify co-regulation modules in between the 3,081 significantly DE genes in at least one pairwise comparison, starting from T7/T0 ratio values calculated for each growth condition (Supplementary Figure S4; Fig. 7a,b). The clustering resulted in four statistically significant clusters with opposite expression profiles in B73 compared to *rmr6* mutant (represented by different background colours in Supplementary Figure 4 and reported in Fig. 7a,b). Three out of the four B73 enriched profiles contained genes up-regulated by stress application at T0, which over-expression was completely transient in all the three stresses (profile 2),or transient in two treatments and maintained at T7 in the remaining one (profiles 4 and 5). On the contrary, the profile 9 includes genes over-expressed at T7 in B73 plants that perceived the combined stress (Fig. 7a).The four *rmr6* enriched profiles enclosed instead either genes in which the stress-induced over-expression was fully maintained ad T7 (profile 10) or genes that were highly expressed at T7 than at T0, with different kinetics depending on the experienced stress (profiles 15, 16 and 17; Fig. 7b).

Comparison of gene expression profiles of DEGs in B73 and *rmr6* confirmed the completely different transcriptional kinetics after recovery from applied stresses between the two genotypes: no co-expressed gene set were statistically significant, while gene assigned to one profile in B73 are included in clusters with unrelated or opposite expression profiles in *rmr6* (Fig. 7c).

### Description, characterization and validation of selected newly annotated transcripts

To more fully investigate the biological role of newly identified transcripts and lncRNAs we selected some of those resulting as differentially expressed in multiple expression analysis for further investigations and expression validation.

**Figure 8:**
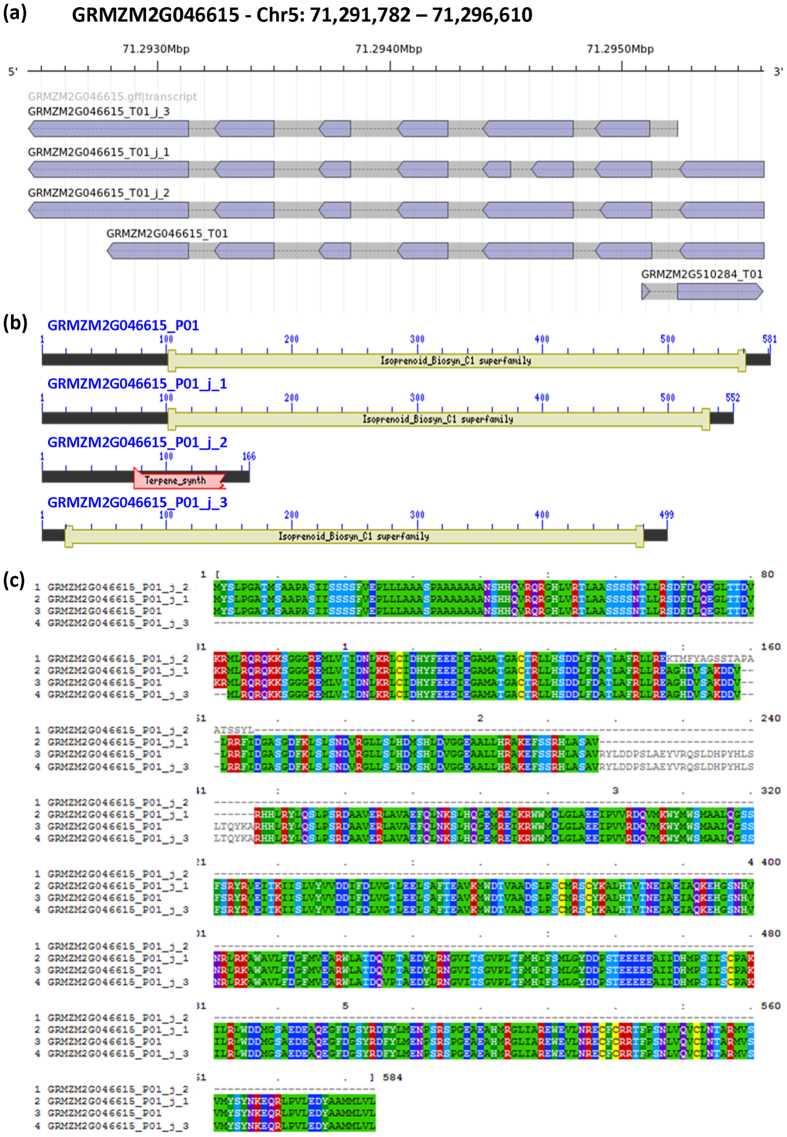
Transcript and protein structures of newly identified isoforms at terpene synthase GRMZM2G046615 locus. (**a**) Isoform structure extracted from the gtf annotation file and plotted with the AnnotationSketch online tool (http://genometools.org/cgi-bin/annotationsketch_demo.cgi). (**b**) Schematic representation of conserved protein domain in the four deduced proteins. (**c)** Amino acid multiple alignment between the four deduced TPS proteins.

GRMZM2G046615, located on chromosome 5, encodes for a putative terpene synthase (TPS) 6-fold and 2.5-fold over-expressed after stresses application in B73 and *rmr6*, respectively (Table 4). TPS enzymes are responsible for the synthesis of the various terpenes, molecules involved in plant defense against biotic and abiotic stresses^48^,^49^. Reference maize annotation included one transcript composed of 7 exons at this locus, while our RABT approach identified three additional isoforms (named GRMZM2G046615_T01_j_1, 2 or 3; Fig. 8). All three newly annotated isoforms include a longer 3′ UTR compared to the reference transcript (Fig. 8a). GRMZM2G046615_T01_j_1 presents an additional intron acceptor/donor site inside the third canonical exon, resulting in an extra exon/intron with the loss of about 30 amino acids from the predicted protein. Despite this extra intron, no deletions could be identified on the protein conserved “Isoprenoid_Biosynthetic” domain (Fig. 8b). In GRMZM2G046615_T01_j_2 an alternative splicing at level of the second exon results in the introduction of a premature stop codon causing a truncated protein, with deleted functional domain (Fig. 8b). Finally, in GRMZM2G046615_T01_j_3 alternative acceptor/donor sites define a different first exon and new start codon, which result in a shorter protein that maintains all the functional domains (Fig. 8b, Supplementary Figure S5). This alternative isoform resulted as being the one with higher expression in all tested samples, with a strong upregulation in both B73 and *rmr6* after stress applications (Table 4).

Although few bases were aligned on the alternative first exon splice site annotated at the GRMZM2G046615_T01_j_3 and a small fraction of mapped reads (15–20%, depending on the library) confirmed this splice junction, 100% of mapped reads confirmed the extra portion of 3′UTR added in the three newly annotated transcripts. This assignment of reads to isoforms together with the Cuffdiff algorithm for isoform estimation could result in biased isoforms RPKM (Reads Per Kilobase Of Exon Per Million Fragments Mapped) estimation. The well-known uncertainty in isoform quantification^50^,^51^ is indeed confirmed by the parallel use of RSEM software, which reported similar mean counts for the four isoforms: all resulted strongly up-regulated by stress application in B73 and lesser in *rmr6* mutant. To confirm the existence of these newly identified splicing variants and to validate their stress-induced gene up-regulation we designed specific primers and used them in RT-PCR for amplification, sequence verification and Q-PCR expression analysis. Given the reduced variations between the reference annotated GRMZM2G046615_T01 transcript and GRMZM2G046615_T01_j_2 and GRMZM2G046615_T01_j_3 novel transcripts, we weren’t able to confirm their existence, while the longer 3′UTR sequence and the additional intron acceptor/donor site inside the third canonical exon of GRMZM2G046615_T01_j_2 were successfully amplified and sequenced (Supplementary Figure S6a). Real-time Q-PCR analysis confirmed the stress-induced up-regulation in both B73 and *rmr6* stressed samples (Supplementary Figure S6b).

**Figure 9:**
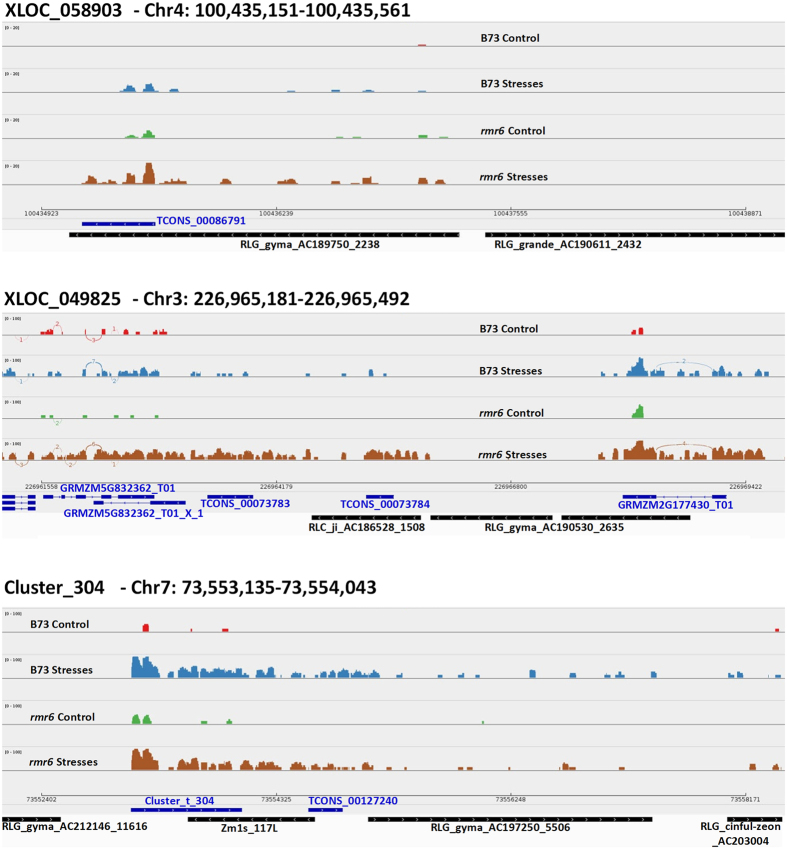
Genome browser view of RNA-Seq reads mapped at Class U newly identified intergenic transcripts. Mapped reads, transcript (blue lines) and repeat (black lines) annotations are reported for three newly identified transcripts differentially expressed in stressed B73 leaves and in *rmr6* mutant. Digital arcs in chromosome 3 snapshot represent the intron-spanning reads that confirm the annotated splice junction.

TCONS_00086791 at locus XLOC_058903, on chromosome 4, is a 410 nt long *de novo* annotated intergenic transcript strongly up-regulated by stress application in B73, while in *rmr6* mutant it is expressed independently of the growth condition (Table 5). This locus corresponds to a Gyma LTR transposable element of the RLG superfamily (Fig. 9a).

Also the isoform TCONS_00073784 (locus XLOC_049825 on chromosome 3) is a newly annotated intergenic transposable-related transcript, but in both genotypes it is not expressed in control condition, being instead activated by osmotic stress application (Table 5). This transcript, together with the close TCONS_00073783 and GRMZM5G832362_T01_X_1 transcripts, presents high similarity with the Ji family of Copia LTR retroelements (Fig. 9b). Based on reads distribution, these three newly annotated transcripts could represent a unique transcriptional product derived from the Ji transposable element.

Finally, Cluster_t_304 is the third representative example of ClassU new intergenic transcripts: this 908 nt long transcript was obtained by the *in silico* fusion of two close TCONS transcripts (Fig. 9c). Cluster_t_304 (and the neighbor TCONS_00127240) is another example of TE-related transcript (Gyma, RLG superfamily of LTR retrotransposon), but differently to TCONS_00086791 and TCONS_00073784, it was expressed in both B73 and *rmr6* under control conditions and further over-expressed by the stress application.

The existence of all these three newly identified intergenic transcripts was confirmed by direct sequencing of RT-PCR amplified fragments while Real-time Q-PCR confirmed their stress-induced expression variation (Supplementary Figure S6b).

Within differentially expressed lncRNAs, GRMZM6G851663_T01 is over-expressed after stress application in both genotypes (Table 6). It is a 421 nt long, intergenic ncRNA, without any matching antisense transcripts, while Blastn analysis revealed a region of partial similarity with GRMZM2G320373_T01, a gene encoding for a bifunctional inhibitor/lipid-transfer protein/seed storage 2S albumin protein, a family of proteins (LPT) with an important action in the defense of plants from different kinds of pathogens and environmental stress^52^. This match is not enough to designate it as the direct target of this lincRNA, anyway it is worth noting that the stress-induced lincRNA up-regulation is strictly associated to the putative target up-regulation in both genotypes. Furthermore, three additional LPT paralogs are stress-induced in B73, while their apparent slight up-regulation in *rmr6* subjected to the same stresses could be caused by to their initial higher expression levels already in control condition (Table 6).

## Discussion

In this work we successfully used a total RNA-Seq approach applied to highly repetitive plant genome such as maize, providing a powerful tool for the in-depth exploration of its transcriptome. Starting from a single tissue we identified more than 25,000 new transcripts, mainly accounting for novel splicing variants at known loci, but also for newly identified intergenic and antisense transcripts. Recently, other authors reported the application of deep sequencing to re-annotate the maize transcriptome of many tissues or developmental stages, achieving the identification of thousands of new transcribed loci or alternative splicing events^15^,^41^,^53^,^54^,^55^. Altogether, these studies confirmed the high discovery potential of RNA-Seq in greatly expanding the maize transcriptome, however, due to the experimental method used for mRNA enrichment, they were limited to polyadenylated transcripts. Our study, based on protocols for rRNA removal from total RNA, provides the first all-inclusive maize transcriptome annotation and an in-depth look at other genomic features, including coding, multiple forms of non-coding RNA and transposable elements.

Applying a specific pipeline the coding potential of the whole maize transcriptome was indeed scanned, identifying 13,387 truly-lncRNAs and 21,624 putative precursors of siRNA, many of which are modulated after stress application and recovery stage, suggesting their important roles in regulating gene expression during abiotic stress response and adaptation. Our pipeline confirmed the 95% of lncRNAs reported in a recent study^41^ and also identified 16,000 additional lncRNA candidates that were previously excluded, most certainly due to the expression filter applied by the authors. Mammalian and plant lncRNAs are normally expressed at lower levels than coding ones and usually show very tissue-, stage-, and individual-specific expression^56^,^57^,^58^. Screening of maize expression atlas strongly supported the actual transcription of at least one quarter of these ncRNA during maize growth and development (for the remaining no expression data are available) and also indicate that more focused genome wide expression studies (at single tissue or specific developmental stage level) are required to truly support their real transcription and to fully address their biological function.

Differential expression analyses identified hundreds of genes modulated by agronomically realistic, long-term stress application, including many lncRNAs and transposons specifically induced by stresses. The number of differentially expressed genes and their expression modulation after stress application and removal appeared very different between B73 and *rpd1-1*/*rmr6* mutant plants, as result of stress-like effect on genome regulation caused by the mutation itself, which activates many stress-related genes even in non-stress growth conditions.

We also discovered that many of the newly identified loci are linked to the de-repression and high expression of TEs belonging to Gypsy and Copia families. TEs activation is detectable in epiregulator mutant background but also in stressed wild type plants, confirming the similar effect of *rmr6* mutation and abiotic stresses on genome regulation. This stress-like transcriptional response caused by the *rmr6* mutation also interests many stress-related genes identified as activated in both *rmr6* unstressed and B73stressed plants, confirming the role of epigenetic mechanisms in plant stress responses. With the recent demonstrated contribution of TEs to the regulation of maize genes in response to abiotic stresses^19^, we can speculate on the existence of highly complex epigenetic interactions between TEs and the transcriptional regulation of their host genome in response to environmental challenges and adaptation.

Combining for the first time a total RNA-Seq approach, with the re- annotation of the transcriptome, its functional annotation, the characterization of its non-coding portion and the identification of gene modulated by osmotic stress applications, this work provides remarkable insights into the genetic and epigenetic mechanisms responsible for maize molecular response to abiotic stresses. This approach applied to further total RNA-Seq analyses conducted on a wide range of tissues and developmental stages will greatly improve the publicly available reference annotations for this important crop species, providing a valuable resource for better addressing the molecular regulation of stress response and the role of the massive amount of transposable elements in maize transcriptional regulation^19^.

## Additional Information

**Accession codes**: The sequenced RNA-Seq data, the final transcriptome annotation (Zea_mays_new_annotation_no_repetitive_v5b.gtf), together with the raw Cuffdiff results for all differential expression tests and the summaries of RPKM values in the different samples for all the annotated genes and transcripts have been deposited at Geo with the accession number GSE71046 (http://www.ncbi.nlm.nih.gov/geo/query/acc.cgi?acc=GSE71046).

**How to cite this article**: Forestan, C. *et al.* Stress-induced and epigenetic-mediated maize transcriptome regulation study by means of transcriptome reannotation and differential expression analysis. *Sci. Rep.* **6**, 30446; doi: 10.1038/srep30446 (2016).

## Supplementary Material

Supplementary Information

Supplementary Data S1

Supplementary Data S2

Supplementary Data S3

Supplementary Data S4

Supplementary Data S5

Supplementary Data S6

Supplementary Data S7

Supplementary Data S8

Supplementary Data S9

## Figures and Tables

**Table 1:** RNA-Seq summary statistics divided by genotypes and replicates.

Summary	Total clean reads	Mapped Reads	Filtered Reads (MAPQ ≥1)
**R1-Non-directional**	589,747,869	577,560,567	433,125,553
** B73**	282,227,233	277,102,278	212,545,639
** *rmr6***	307,520,636	300,458,289	220,579,914
**R2-Directional**	617,597,121	609,264,774	463,578,769
** B73**	302,169,747	299,108,987	225,729,599
** *rmr6***	315,427,374	310,155,787	237,849,170
**TOTAL**	1,207,344,990	1,186,825,341	896,704,322
** B73**	584,396,980	576,211,265	438,275,238
** *rmr6***	622,948,010	610,614,076	458,429,084

Total clean reads refer to the number of reads after quality trimming and contaminant filtering, while filtered reads represent the number of reads mapped to the maize B73 AGPv3 reference genome with a Mapping quality score of at least 1, after the removal of PCR duplicates. Statistics for all 32 sequenced libraries are reported in Supplementary Data S1.

**Table 2:** Summary of lncRNAs identification and classification.

Transcript Class	Coding	pot-lncRNA	lncRNA	siRNA-prec	TEs
Equal	102,299	31,364	11,815	18,526	1,023
Class J	19,796	1,603	386	1,181	36
Class U	995	1,669	625	1,005	39
Class O	627	363	96	257	10
ClassX	103	288	116	164	8
Previously annotated ncRNAs	517	864	349	491	24
Total	124,337	36,151	13,387	21,624	1,140

The number of coding and potential long non-coding RNAs is reported for each transcript annotation class. Pot-lncRNAs were further classified in truly lncRNA, siRNA, shRNA precursors or TE-derived transcripts.

**Table 3:** TE families classification of up-regulated transcripts.

FAMILY	Stresses vs Control B73		Stresses vs Control rmr6		rmr6 vs B73	
RLG-Gypsy	28	40.0%	21	48.8%	42	35.9%
RLC-Copia	6	8.6%	7	16.3%	35	29.9%
RLX-Unknown LTR	9	12.9%	2	4.7%	12	10.2%
RIL-L1	1	1.4%	0	0.0%	0	0.0%
DTA-hAT	13	18.6%	3	7.0%	4	3.4%
DTC-CACTA	4	5.7%	4	9.3%	10	8.5%
DHH-Helitron	2	2.9%	3	7.0%	1	0.9%
DTM-Mutator	3	4.3%	0	0.0%	5	4.3%
DTH-PIF/Harbinger	3	4.3%	2	4.7%	5	4.3%
DTT-Tc1/Mariner	1	1.4%	1	2.3%	1	0.9%
RST-tRNA	0	0.0%	0	0.0%	2	1.7%
**Total TE-related transcripts**	**70**	**6.4%**	**43**	**13.9%**	**117**	**18.2%**
Total DE transcripts	1,094		310		622	

For each pairwise comparison, the number and relative percentage of up-regulated transcripts identified as TE-related and assigned to each TE superfamily are reported. About 20% of the transcripts upregulated in mutant background are TE-related, while lower percentages are induced by stresses application. Gypsy and Copia class I LTR families are over-represented in all three sets of upregulated transcripts, with also an interesting percentage of transcripts ascribable to the DTA/hAT family of classII DNA transposon induced by stresses application in B73.

**Table 4:** RPKM values estimated for isoforms at GRMZM2G046615 locus.

ID	Status	B73 Control	B73 Stresses	log2 FC	rmr6 Control	rmr6 Stresses	log2 FC
GRMZM2G046615	OK	7.68	45.13	2.55	10,14	25.32	1.32
GRMZM2G046615_T01	NOTEST	0	0.12		0	0.12	
GRMZM2G046615_T01_j_1	NOTEST	0	0.14		0.068	0.04	
GRMZM2G046615_T01_j_2	NOTEST	0	0.14		0	0	
GRMZM2G046615_T01_j_3	OK	7.67	44.73	2.54	10.08	25.15	1.32

RPKM (Reads Per Kilobase per Million mapped reads) are reported for the gene GRMZM2G046615 and for the four isoforms identified at this locus. “NOTEST” in the Status column indicate there were not enough aligned reads for testing the differential expression.

**Table 5:** RPKM values estimated for newly annotated TCONS transcripts.

Isoform	Status	B73 Control	B73 Stresses	log2 FC	rmr6 Control	rmr6 Stresses	log2 FC	log2FC rmr6/B73
TCONS_00086791	OK	0	1.08	6.77	1.26	1.53	0.28	6.97
TCONS_00073784	OK	0	1.14	6.84	0	3.27	8.36	0
Cluster_t_304	OK	0.94	5.30	2.47	1.08	4.07	2.17	0.2

RPKM (Reads Per Kilobase per Million mapped reads) are reported for some of the newly annotated TCONS transcripts.

**Table 6:** RPKM values estimated for lncRNA GRMZM6G851663_T01 and its presumptive targets.

Isoform	Status	B73 Control	B73 Stresses	log2 FC	rmr6 Control	rmr6 Stresses	log2 FC
GRMZM6G851663_T01	OK	16.88	65.19	1.95	29.35	85.30	1.54
GRMZM2G320373_T01	OK	4.26	26.37	2.62	8.62	30.94	1.84
GRMZM2G004909_T01	OK	17.25	62.71	1.86	80.40	114.96	0.51
GRMZM2G137329_T01	OK	70.10	199.62	1.51	108.76	194.91	0.84
GRMZM2G170044_T01	OK	10.59	24.52	1.21	17.12	20.30	0.24

RPKM (Reads Per Kilobase per Million mapped reads) are reported for lncRNA GRMZM6G851663_T01 and its presumptive targets.
