# Supplementary material for: Stress-induced and epigenetic-mediated maize transcriptome regulation study by means of transcriptome reannotation and differential expression analysis
Source: Sci Rep. 2016 Jul 27;6:30446. doi: 10.1038/srep30446 (PMC4962059; doi:10.1038/srep30446)
Supplement: Supplementary Information [file srep30446-s1.pdf]

Stress-induced and epigenetic-mediated maize transcriptome regulation study by means of transcriptome reannotation and differential expression analysis

Cristian Forestan[1*], Riccardo Aiese Cigliano[2], Silvia Farinati[1], Alice Lunardon[3], Walter Sanseverino[2] and Serena Varotto[1]

Supplementary Tables S1-S4

Supplementary Figures S1-S6

Supplementary Data Description

Supplementary Table S1: Summary of differential expression analyses results

Number of genes differentially expressed (log2 fold change ratio ≥ |1| and FDR- adjusted p value ≤ 0.05) in each pairwise comparison divided by their annotation class.

	**Stresses vs Control B73**		**Stresses vs Control rmr6**		**rmr6 vs B73**	
	Up-regulated	Down-regulated	Up-regulated	Down-regulated	Up-regulated	Down-regulated
**DE Genes**	1,763	69	588	48	799	318
**Reference**	1,708	63	555	48	549	293
**Class O**	7	1	2	0	7	3
**Class X**	1	0	2	0	13	1
**Class U**	47	5	29	0	230	21

	**Stresses vs Control B73**		**Stresses vs Control rmr6**	
	Up-regulated	Down-regulated	Up-regulated	Down-regulated
**DE Genes**	462	40	845	54
**Reference**	451	36	810	51
**Class O**	2	0	8	0
**Class X**	0	0	2	0
**Class U**	9	4	25	3

Supplementary Table S2: Summary of differential expression analyses results

Number of transcripts differentially expressed (log2 fold change ratio ≥ |1| and FDR- adjusted p value ≤ 0.05) in each pairwise comparison subclassified based on their coding potential.

	Stresses vs Control B73		Stresses vs Control rmr6		rmr6 vs B73	
	Up-regulated	Down-regulated	Up-regulated	Down-regulated	Up-regulated	Down-regulated
**DE Transcripts**	1,094	32	310	30	622	140
**Coding**	965	21	217	21	350	104
**siRNA-precs**	78	7	66	5	189	25
**lncRNAs**	51	4	27	4	83	11

	Stresses vs Control B73		Stresses vs Control rmr6	
	Up-regulated	Down-regulated	Up-regulated	Down-regulated
**DE Transcripts**	188	11	510	35
**Coding**	166	5	429	30
**siRNA-precs**	13	4	46	1
**lncRNAs**	9	2	35	4

Supplementary Table S3: List of the 44 genes with stress-responsive ontology upregulated at the same time in *rmr6* control conditions and B73 stressed samples.

Gene ID	Annotation
GRMZM2G004160	Chemocyanin
GRMZM2G010740	Uncharacterized protein
GRMZM2G021406	Lysine decarboxylase-like protein
GRMZM2G025833	Beta-amylase
GRMZM2G028535	Delta 1-pyrroline-5-carboxylate synthetase
GRMZM2G043095	Uncharacterized protein
GRMZM2G044383	Glutathione S-transferase GST 30
GRMZM2G044851	Nitrate transporter 1.5
GRMZM2G049349	Peptidase, M50 family
GRMZM2G052344	Uncharacterized protein
GRMZM2G053669	Asparagine synthetase
GRMZM2G054115	Uncharacterized protein
GRMZM2G054803	Uncharacterized protein
GRMZM2G058173	Undecaprenyl pyrophosphate synthetase
GRMZM2G059836	Farnesylated protein 2
GRMZM2G074759	Putative AMP-dependent synthetase and ligase superfamily protein
GRMZM2G076844	Oxidative stress 3
GRMZM2G078465	Uncharacterized protein
GRMZM2G087507	Aldose reductase
GRMZM2G088501	Uncharacterized protein
GRMZM2G092474	Uncharacterized protein
GRMZM2G099454	Uncharacterized protein
GRMZM2G103945	Aquaporin TIP4-1
GRMZM2G117878	Uncharacterized protein
GRMZM2G125032	Beta-1,3-glucanase

GRMZM2G136960	Stress enhanced protein 2
GRMZM2G139300	Beta-fructofuranosidase, cell wall isozyme
GRMZM2G150906	Raffinose synthase family protein
GRMZM2G154735	HVA22-like protein e
GRMZM2G158394	Extracellular ribonuclease LE
GRMZM2G162056	GNAT-transcription factor 13
GRMZM2G165919	Galactinol synthase 3
GRMZM2G169149	WRKY62 transcription factor
GRMZM2G176085	Uncharacterized protein
GRMZM2G181000	Uncharacterized protein
GRMZM2G374302	Arginine decarboxylase
GRMZM2G395526	Putative protein
GRMZM2G450233	Uncharacterized protein
GRMZM2G453805	Chitinase A
GRMZM2G455909	Uncharacterized protein
GRMZM5G801627	Uncharacterized protein
GRMZM5G809218	Putative protein
GRMZM5G891656	Monooxygenase
GRMZM5G893912	Uncharacterized protein

Supplementary Table S4: List of primers used in this study.

Gene	Primer Fw	Primer Rev	Application
**Cluster_t_304**	GGT GTG AGG AGT ACT CAA AGT GAT TC	CAC ACA CTT CGG AGA TAA GAG CGG CAG	Sequence amplification and Q-PCR
**TCONS_00073784**	GTC AGA GCT GTC AGA AAA GTC ATG TG	GTG ATT AAG AGA GAG AGT GTG GGG G	Sequence amplification and Q-PCR
**TCONS_00086791**	CCT AAT TCC AGC TTG CCT GTT CTT G	CCA TAA CCT AAA CTG GTT GAT GAA GGG	Sequence amplification and Q-PCR
**GRMZM2G046615**	GTC GTA CAG GGA CTT CTA CCT CAT G	CTC CTA CAG GGG CCA TAT TAC ATT GC	Sequence amplification and Q-PCR
**GRMZM2G046615_j_1**	CTC ACA GCT ATA CAT CGG CGC ATC G	GTA GCG GAG GTG ATG CCT GAC GG	Sequence amplification
**GRMZM2G046615_j_3**	GTA CGT GCG GCA GTC CCT GGA C	ATG AGG TAG AAG TCC CTG TAC GAC	Sequence amplification
**GAPC2**	AAT GGC AAG CTC ACT GGC	CTG TCA CCG GTG AAG TCG	Q-PCR housekeeping

Supplementary Figure S1: Flow-chart of the overall experimental design and RNA-Seq data analysis.

The scheme summarize the whole experimental design, starting from plant growing condition, stress application and samples collection to NGS Illumina sequencing and software employed for transcriptome re-annotation, characterization and differential expression analysis.

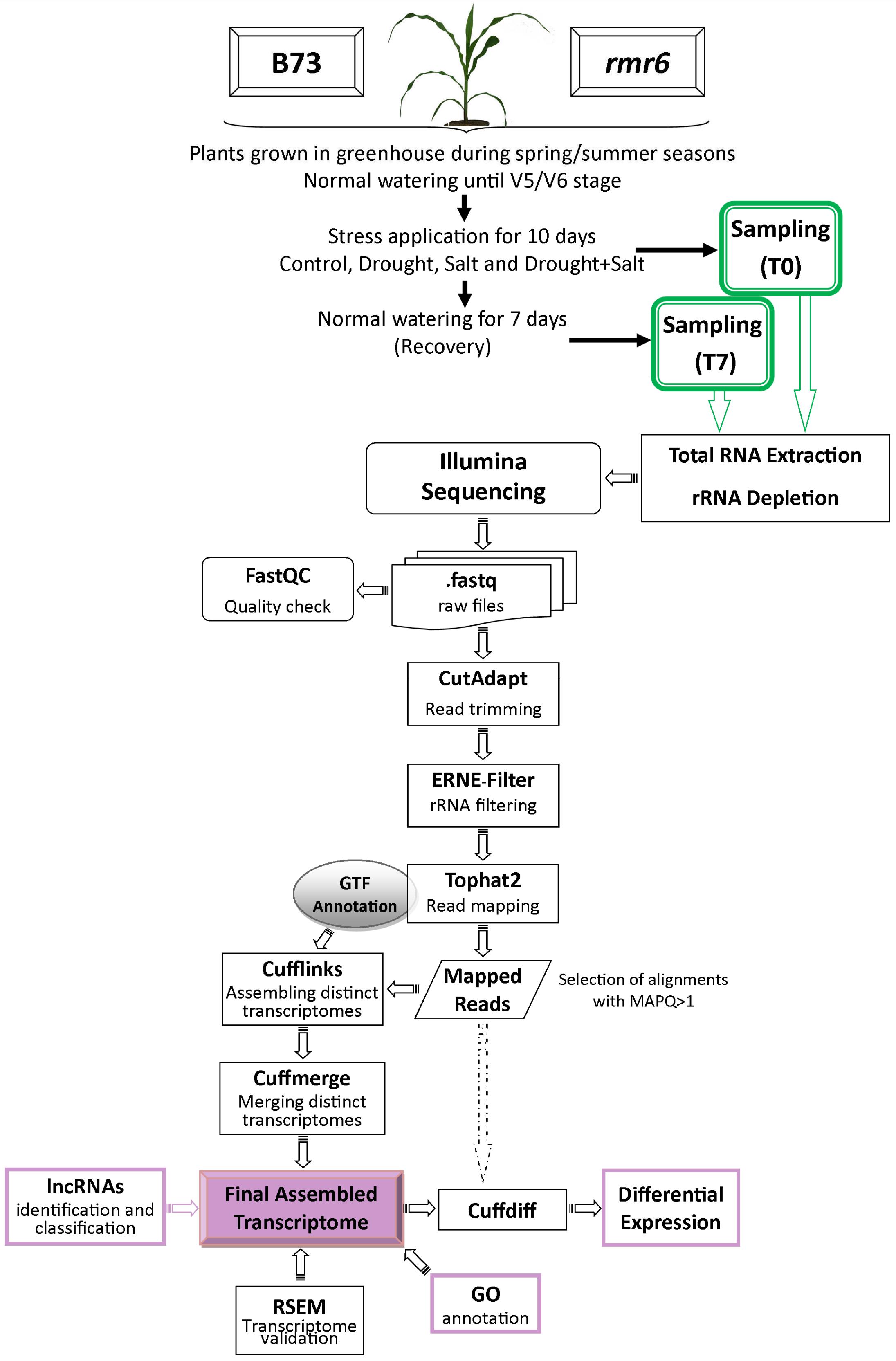

Supplementary Figure S2: Histogram comparing GO terms newly assigned to *Z. mays* transcripts to EnsemblPlants/Biomart reference annotation

The results obtained with WEGO annotation plotting tool are summarized for biological process, cellular component and molecular function categories. The right Y-axis represents the number of transcripts and the left Y-axis shows the percentage of total transcripts. Red bars represent values corresponding to new GO annotation while blue ones stand for the reference annotation.

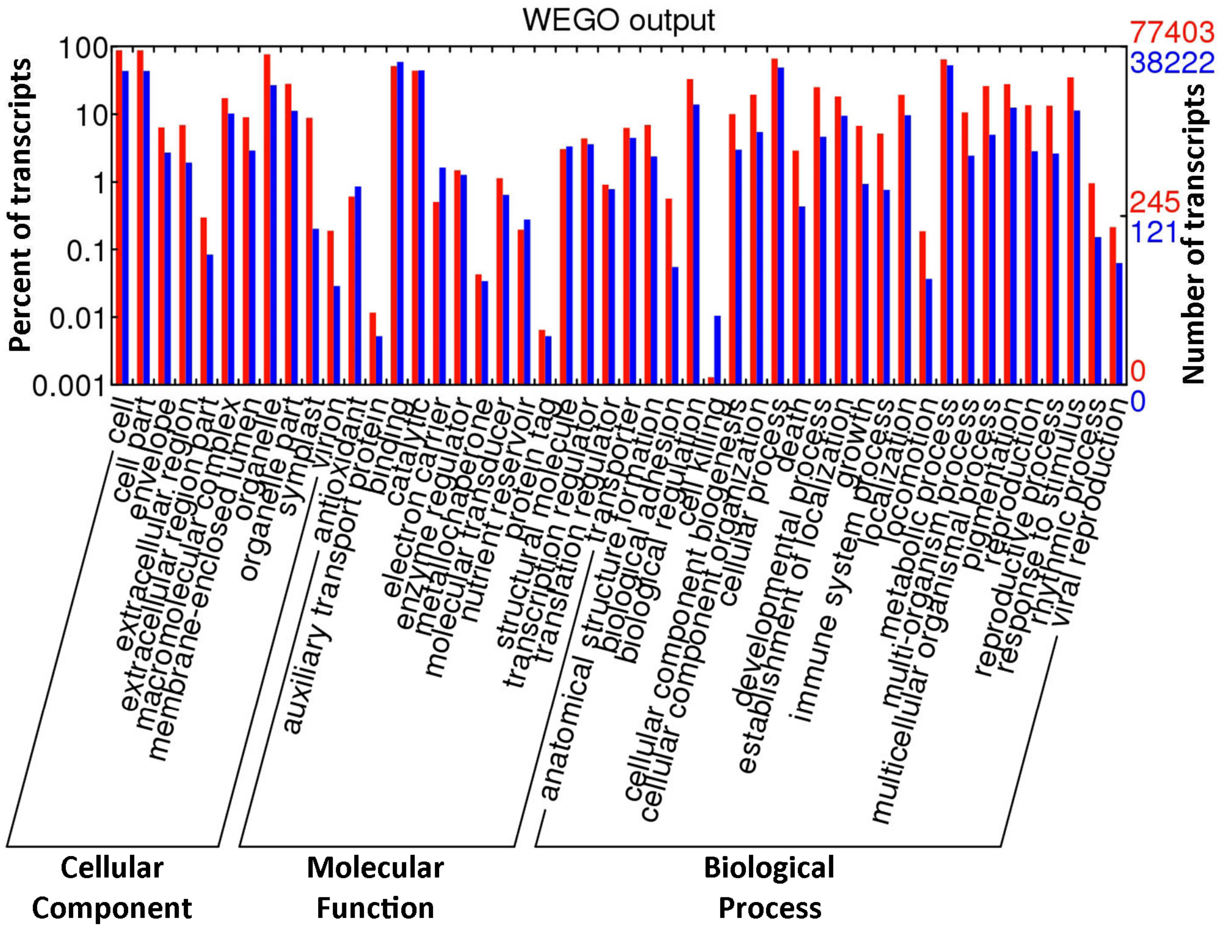

Supplementary Figure S3: Comparison of DEG profiles under different osmotic stresses

Expression profile and clusters of DEGs obtained by the STEM clustering of B73 (red) and *rmr6* (blue) subjected to the three different stress conditions. Each box corresponds to one of the model expression profiles and the numbers indicated profile unique ID (upper-left corner) and number of genes falling into the cluster (bottom-left corner). Clusters were ordered according to the number of genes, while significantly enriched profiles (that have a statistically significant number of genes assigned compared to the number of genes expected based on the permutation test) were represented by different background colors (see also Figure 5a-b).

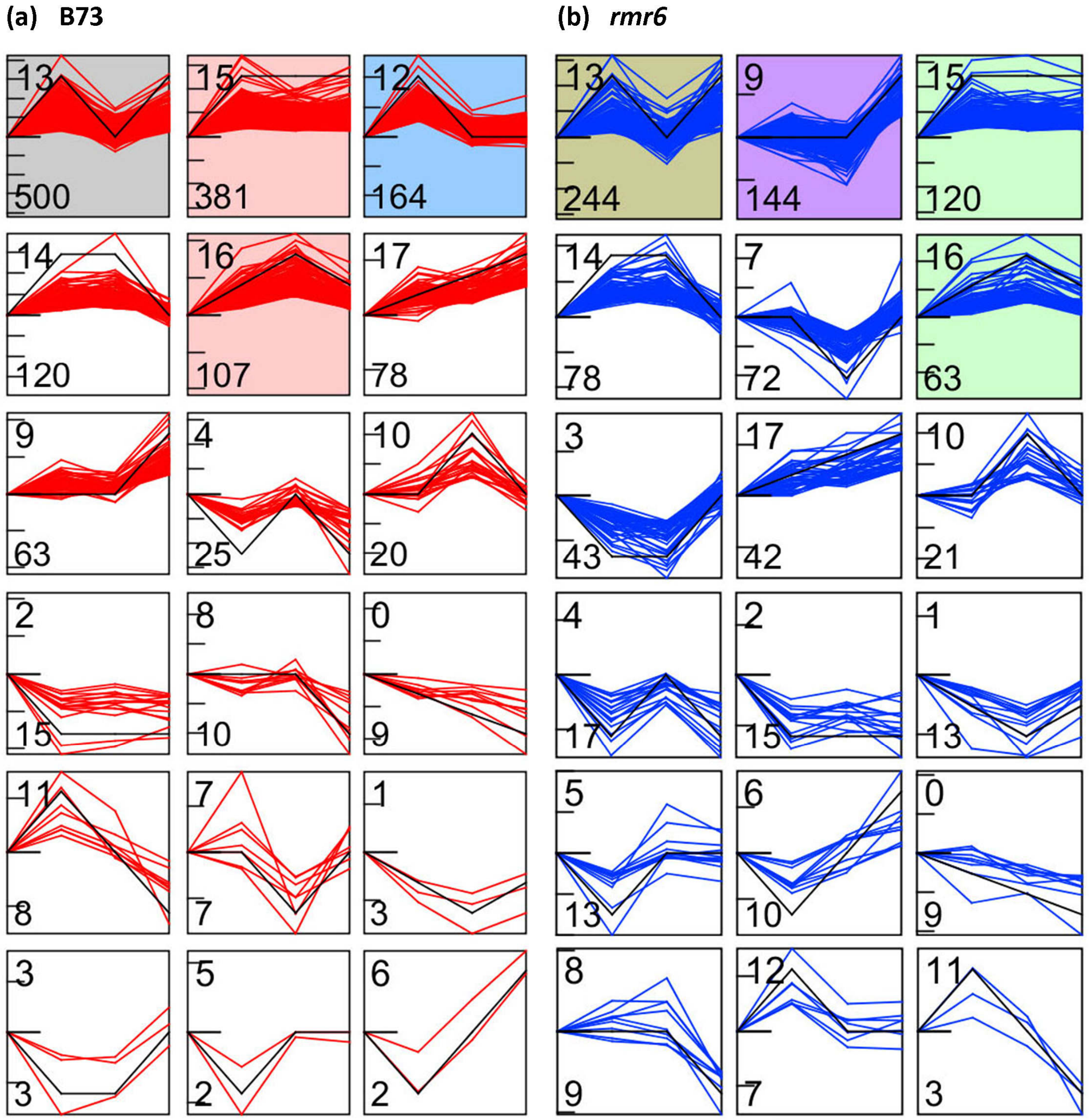

Supplementary Figure S4: Comparison of DEG profiles under different osmotic stresses and recovery stage

Expression profile and clusters of DEGs obtained by the STEM clustering of B73 (red) and *rmr6* (blue) subjected to the three different stress conditions and the recovery stage, plotting the to the log2 T7/T0 expression ratio of each DE gene for the three analyzed stresses. Each box corresponds to one of the model expression profiles and the numbers indicated profile unique ID (upper-left corner) and number of genes falling into the cluster (bottom-left corner). Clusters were ordered according to the number of genes, while significantly enriched profiles (that have a statistically significant number of genes assigned compared to the number of genes expected based on the permutation test) were represented by different background colors (see also Figure 7a-b).

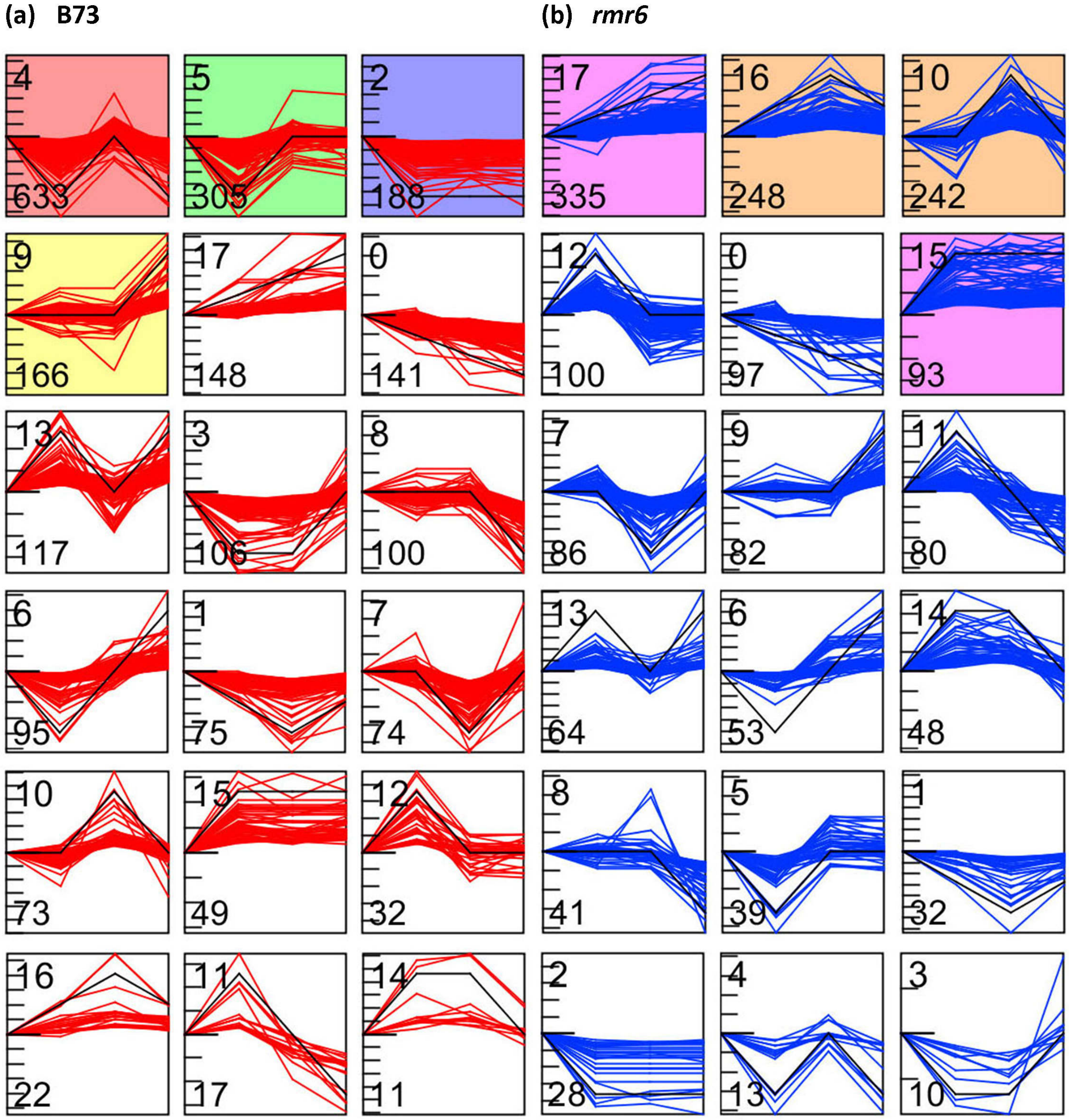

Supplementary Figure S5: Multiple alignment between maize and Arabidopsis terpene synthase proteins.

The N-terminal domain, partially missing in GRMZM2G046615_T01_j_3, presents high variability in terms of length and composition between the TPS proteins of maize and Arabidopsis, compared to the more conserved central and C-terminal regions.

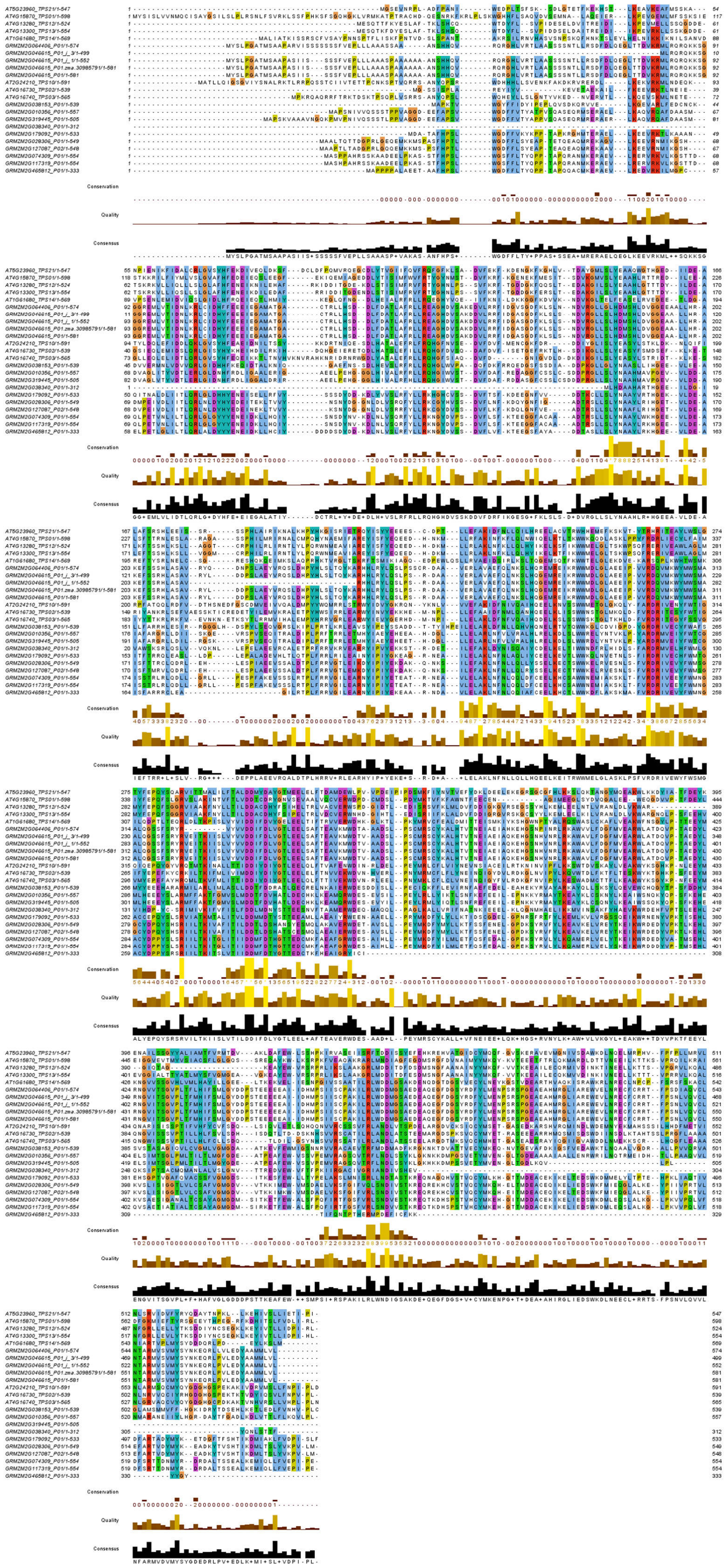

Supplementary Figure S6: Sequence and expression validation of newly identified transcripts

a) Multiple alignment between GRMZM2G046615 genomic sequence, GRMZM2G046615_T01 reference annotated transcripts and GRMZM2G046615_T01_j_1 and GRMZM2G046615_T01_j_3 newly identified new isoforms, showing primers used for sequence amplification and sequencing (yellow and blue arrows) and sequence-validated features. Arrowhead in GRMZM2G046615_T01_j_2 marks the premature stop codon. **b**) Expression validation by Q-PCR for the selected stress-induced genes.

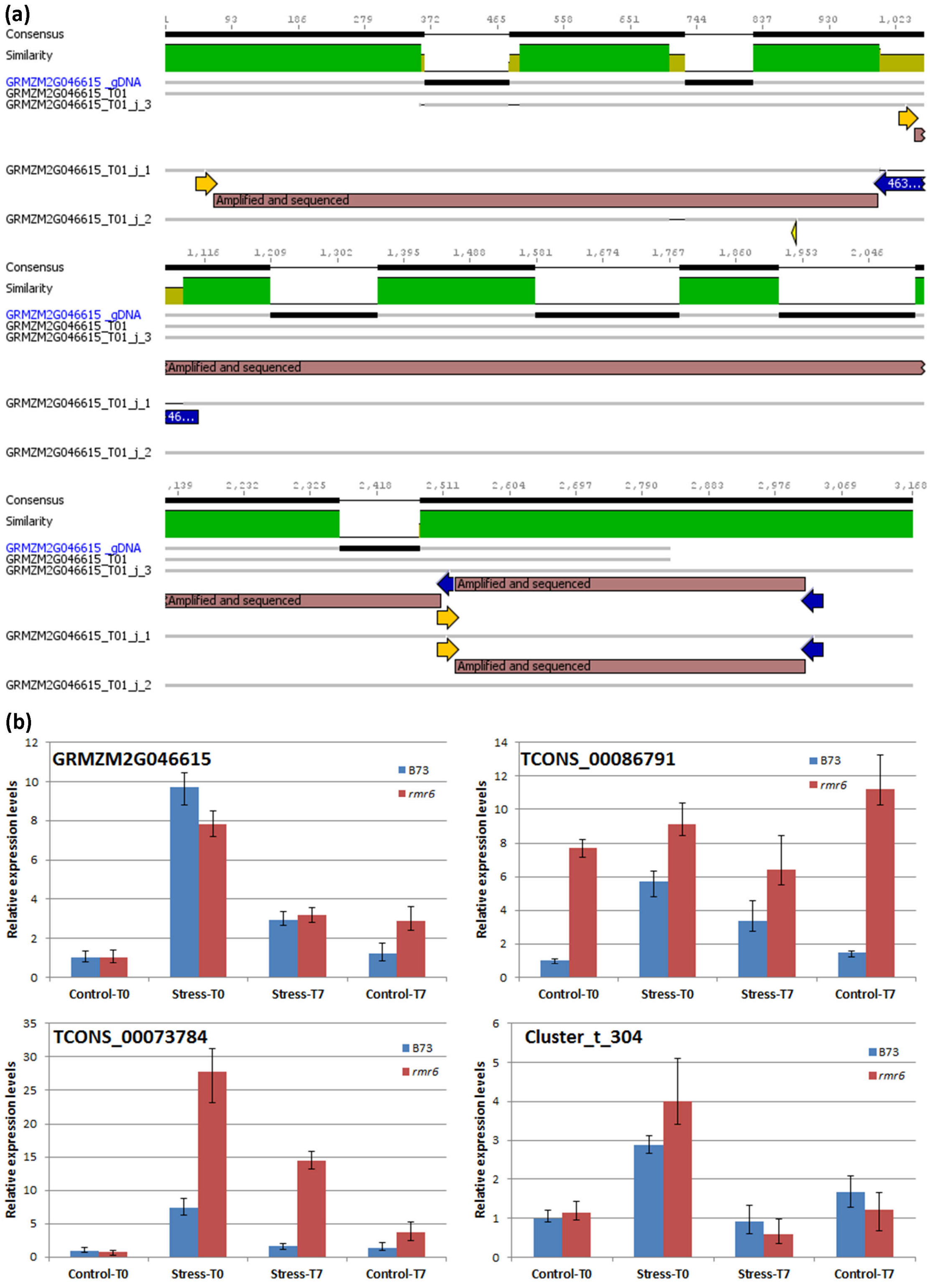

Supplementary Data S1: RNA-Seq summary statistics relative to the 32 sequenced libraries

Number of total sequenced reads, clean reads (after quality trimming and contaminant filtering) and filtered reads (read mapped to the maize B73 AGPv3 reference genome with a Mapping quality score -MAPQ- of at least 1, after the removal of PCR duplicates) and related statistics for each of the sequenced libraries.

Supplementary Data S2: Blast2GO GO annotation file

Comma separated Annot file reporting the Blast2GO summary: specific GO terms were successfully assigned to 77,403 transcripts.

Supplementary Data S3: Functional enrichment of GO-terms analysis in newly identified transcripts

Overrepresentation of Gene Ontology terms was analyzed in each newly annotated Cufflinks transcript class with Ontologizer software. Raw and Revigo summarized results are reported for each transcript class. Statistically enriched terms (adjusted p-value < 0.1) are reported in bold format.

Supplementary Data S4: Newly identified antisense/sense loci pairs

Each newly antisense Class X locus was coupled with its sense reference locus. Transcript information and putative annotation are reported for each sense/antisense pair.

Supplementary Data S5: ncRNAs classification summary

Characteristics of all the putative ncRNAs identified.

Supplementary Data S6: Transcription evidences for a subset of pot- and truly-lncRNAs

Expression levels (expresses as FPKM) retrieved from a recently published expression atlas (Stelpflug et al., 2016) and from the qTeller tool (http://www.qteller.com/) for the subset of lncRNAs and non-coding siRNA precursors enclosed these databases.

Supplementary Data S7: Complete Cuffdiff results of the three differential expression tests at T0

Statistically differentially expressed genes and transcripts identified by Cuffdiff for the three differential expression analyses performed at T0.

Supplementary Data S8: FPKM values in B73 and *rmr6* samples

Summary of FPKM values in the different samples for subset of transcripts differentially expressed in B73 in response to osmotic stresses.

Supplementary Data S9: Complete Cuffdiff results of the two differential expression tests at T7

Statistically differentially expressed genes and transcripts identified by Cuffdiff for the three differential expression analyses performed at T7.
